# Activity Coefficients of HCl in Solutions Related to “Tris” Buffers in Artificial Seawater. III. Tris Buffer + NaCl + H_2_O, from 0.2 to 3.25 mol kg^–1^ Ionic Strength and from 5 to 45 °C

**DOI:** 10.1021/acs.jced.5c00450

**Published:** 2025-12-05

**Authors:** Frank Bastkowski, Beatrice Sander, Simon L. Clegg

**Affiliations:** † 39428 Physikalisch-Technische Bundesanstalt (PTB), Bundesallee 100, Braunschweig 38116, Germany; ‡ School of Environmental Sciences, University of East Anglia, Norwich NR4 7TJ, United Kingdom

## Abstract

The substance Tris­(2-amino-2-hydroxymethyl-1,3-propanediol, CAS 77-86-1), and its protonated form TrisH^+^, are used in the preparation of “total” pH buffers in artificial seawater media. Here, as part of a series of studies, we present Harned cell measurements of potentials in solutions containing equimolal Tris and TrisHCl (hence TrisH^+^), and also NaCl which is the major constituent of artificial seawater. The methods of preparation of the hydrogen and chloride electrodes are described. The data contribute to the development of a chemical speciation model of the buffer solutions in which solute activity coefficients are calculated using the Pitzer equations. Such a model is required in order to quantify the effects of composition change, convert the total pH to other scales, and to address metrological requirements for traceability. The results are expressed in terms of an acidity function and are compared to previous measurements at 25 °C, including equivalent values for artificial seawater media, and also to calculations using a preliminary model. Agreement is good, and the small differences found between data and model predictions are likely due to offsets in the measured potentials, and uncertainties in some of the Pitzer model parameters and the TrisH^+^ dissociation constant.

## Graphical abstract

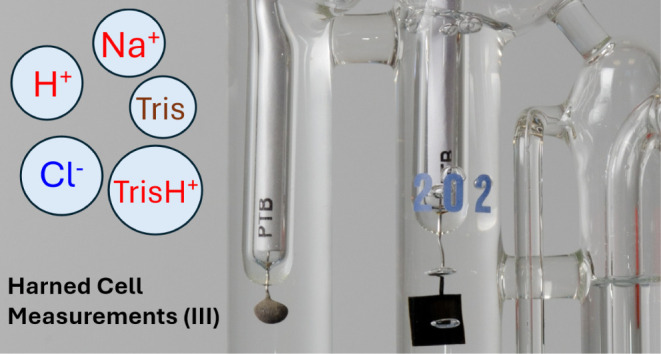

## 1 Introduction

Artificial seawaters, and natural seawater, consist of about 90 mol % Na^+^ and Cl^–^ ions, plus smaller amounts of Mg^2+^, SO_4_^2–^, Ca^2+^, and K^+^. The seawater total hydrogen ion pH scale was established from measurements of cell potentials of solutions of artificial seawater acidified with HCl,^1^ and similar solutions containing equimolal Tris and its conjugate acid TrisH^+^ as a pH buffer.^2^ The development of a chemical speciation model of these buffer solutions, yielding molalities and activities of solute species for a range of salinities and temperatures, and hence total pH, was suggested by Dickson et al.^3^ and has several potential benefits. These include the extension of the scale to a wider range of temperatures and salinities, conversion to other forms such as “free” pH^4^ and conventional pH,^5^ and improved metrological traceability.^3,6^

Clegg et al.^6^ developed a draft model of Tris buffer in artificial seawater (for 25 °C only), using the Pitzer equations^7^ for the calculation of activity coefficients, and tabulated the unknown Pitzer interaction parameters that new thermodynamic data are needed to quantify. In two previous studies, Maksimov et al.^8,9^ have determined mean activity coefficients of HCl in aqueous HCl-TrisHCl and HCl-NaCl-TrisHCl solutions, and the value of an acidity constant in the Tris buffer-NaCl solutions, from measured potentials of Harned cells which yield activity products of H^+^ and Cl^–^. This study is the third of a series which involves the national metrology institutes of Japan, Germany (hereinafter PTB), and the USA. Here we present results of measurements of electrochemical potentials of equimolal Tris buffer in aqueous NaCl solutions over a range of temperatures and ionic strengths, as a further step toward developing a model of the pH buffer. The results are compared with available data for these solutions. The application of the Pitzer model to calculate the speciation and cell potentials, using currently available interaction parameters and equilibrium constants, is also reviewed. Elements of the model that need improvement are identified and recommendations for further work are made.

## 2 Experimental Methods

Activity products of H^+^ and Cl^–^ ions were determined from measurements of the potential difference of the following electrochemical cell:

$${\mathrm{Pt}}_{\left(s\right)},H_{2\left(g\right)}\left(1\;\mathrm{atm}\right)\left|H^{+},{\mathrm{Cl}}^{‐}\;\mathrm{in}\;\mathrm{aq}.\;\mathrm{soln}.\,\right|{\mathrm{Ag}}_{\left(s\right)}/{\mathrm{AgCl}}_{\left(s\right)}$$ A

where the solutions in this study contain stoichiometric equimolal TrisHCl and Tris (i.e., Tris buffer) in aqueous NaCl. In the buffer solutions the H^+^ content is determined by the acid–base equilibrium between the TrisH^+^ ion and Tris,^10^ which yields a slightly alkaline solution. The potential, *E* (V), of cell A is given by the following expression:

1 $$E=E^{0}-\left(RT/F\right)\cdot \ln\left(aH^{+}\cdot aCl^{‐}\right)$$

where *E* ^0^ (V) is the standard potential of the cell at the temperature *T* (K) of interest, *R* (8.31446 J mol^–1^ K^–1^) is the gas constant, *F* (96485.332 C mol^–1^) is Faraday’s constant, and prefix *a* denotes activity. The activity product of the H^+^ and Cl^–^ ions can also be written *m*H^+^·*m*Cl^–^·γ_HCl_^2^, where prefix *m* indicates molality and γ_HCl_ is the mean activity coefficient of H^+^ and Cl^–^ ions in the solution.

A schematic of a Harned cells used at PTB is shown in Figure 1. A flow of dry hydrogen gas at a rate of 8 cm^3^ min^–1^ first passes through two presaturators containing an aqueous solution of the same composition as that being measured. The gas flow next passes into the half-cell of the U-shaped measurement compartment containing the platinum hydrogen electrode, and bubbles through the solution and exits at the top. This half-cell is connected, with a glass capillary tube, to the other half-cell which contains the reference silver – silver chloride electrode.

**Figure 1:**
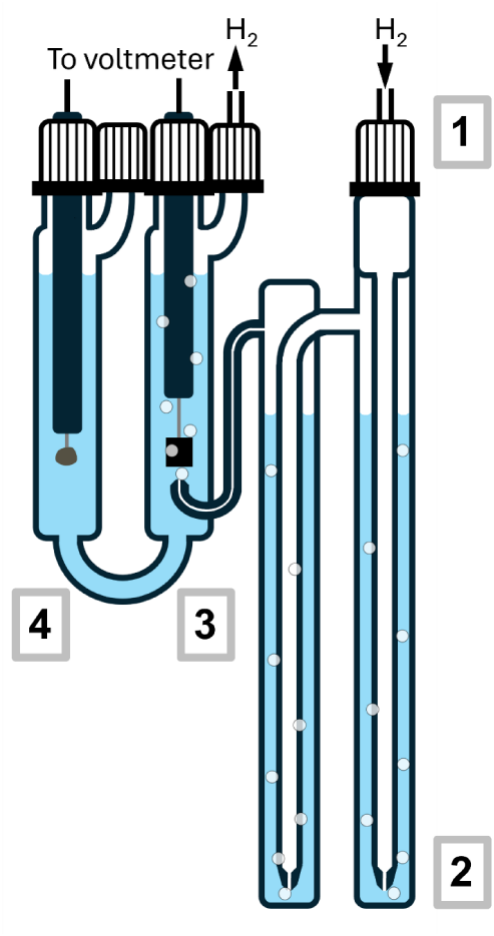
Harned Cell. A flow of dry hydrogen enters the apparatus at the top right (1), and then passes through the pair of presaturators (2), and into the half-cell containing the solution being measured and a platinum hydrogen electrode (3). The gas flow exits at the top of that half-cell, which is is connected, via a glass capillary tube, to half-cell (4) which contains the same solution and a silver – silver chloride electrode. The whole cell is immersed in a water bath for temperature control.

The preparation of hydrogen and reference electrodes is described by Bates,^11^ and the specific procedures used at PTB are summarized in the Supporting Information. The equivalent procedures used at the National Metrology Institute of Japan in our other recent studies are described by Maksimov et al.^8^

A set of 12 Harned cells is used for each measurement run. The cells are immersed in a temperature-controlled Lauda Proline PV36 thermostatic bath/circulator, operating with a Lauda DLK 45 through-flow cooler. The temperature was measured with a ASL-WIKA CTR5000 thermometer together with 4 PT100 temperature probes. Hydrogen gas of 6.0 quality (“Hydrogen 6.0”) was obtained from Linde Gas. Cell potentials were measured with a Keysight 3458A multimeter (Keysight Technologies). The atmospheric pressure was measured with a Setra 370 pressure meter (Setra Systems Inc., Boxborough, USA).

### 2.1 Solution Compositions and Preparation

The Tris buffer solutions contain stoichiometric molalities of 0.04 mol kg^–1^ Tris and TrisH^+^ cation in an NaCl medium with total ionic strengths (*I*) of 0.2 to 3.25 mol kg^–1^ which in these solutions is the same as the total chloride molality.

The chemicals used in the preparation of the solutions are listed in Table 1. The solid Tris was stored at room temperature and used directly from the sealed bottles supplied by the manufacturer, without additional drying, to prepare a Tris stock solution of 2 mol kg^–1^ molality. A hydrochloric acid stock solution of ∼ 1 mol kg^–1^ molality was prepared by dilution of high purity hydrochloric acid (Titrisol, Merck company) with ultrapure water. The purity of the sodium chloride (Suprapur, Merck) was coulometrically determined by the Slovak Metrology Institute after drying at 110 °C for 2 h, and a 4 mol kg^–1^ stock solution was prepared. The measurement solutions were gravimetrically prepared from the stock solutions and ultrapure water after buoyancy correction, assuming an average air density of 1.2 kg m^–3^. The prepared solutions covered the chloride molality and ionic strength range 0.2–3.25 mol kg^–1^, and contained equal stoichiometric molalities of 0.04 mol kg^–1^ of Tris and TrisH^+^ (from the neutralization of half of the total Tris by HCl).

**Table 1:** Chemicals Used in This Study

Chemical	CAS Registry	Molar mass (g)	Supplier or source	Notes
Tris^a^	77-86-1	121.135	National Institute of Standards and Technology (NIST), SRM 723e	A purity of 100% was assumed
HCl	7647-01-0	36.4609	Merck, Titrisol	HCl content was coulometrically quantified
				1. For the preparation of the measurement solutions: *m*HCl = 0.994334 ± 0.000153 mol kg^–1^
				2. For the measurement of the standard potential with 0.01 mol kg^–1^ HCl: *m*HCl = 0.01001602 ± 1.75×10^–6^ mol kg^–1^
H_2_O	7732-18-5	18.0153	Milli-Q Ultra Pure Water System (Merck)	
NaCl	7647-14-5	58.4428	Merck Suprapur	Purity coulometrically quantified by the Slovak Institute of Metrology (SMU) to be 99.892% ± 0.040%

a 2-Amino-2-(hydroxymethyl)­propane-1,3-diol, C_4_H_11_NO_3_.

The dilute aqueous HCl required to determine standard cell potentials was prepared by dilution of high purity hydrochloric acid (Titrisol, Merck company) with ultrapure water. The molality was determined by coulometry to be 0.01001602 ± (1.75 × 10^–6^) mol kg^–1^.

### 2.2 Measurements

Cell potentials were measured from 5 to 45 °C for all solutions. Identifiers for the individual cells used, the chloride or HCl molalities of the solutions, and the dates of measurement, are listed in Table 2. The measurements were carried out in two series: from 0.2 to 1.5 mol kg^–1^ ionic strength solutions in late 2020, and the higher molality solutions (plus a remeasurement of 0.2 and 0.4 mol kg^–1^ solutions) in January 2021. Measurements of 0.01 mol kg^–1^ HCl for the determination of standard potentials were made separately in each series. In the dilute solutions usually measured at PTB the criterion of stability of the cell potential – indicating that a measurement can be made – is a voltage drift not exceeding 50 μV h^–1^. This same criterion was applied in this study for all solutions measured.

**Table 2:** Cell Identifiers and Dates of Measurements

Cell Number	Series	*m*Cl^–^ ^a^ (mol kg^–1^)	Date	Cell Number	Series	*m*HCl^b^ (mol kg^–1^)	Date
3	1	0.20	02/12/20	1, 2, 10, 11, 12	1	0.010016	02/12/20
4	1	0.40	02/12/20	1, 11, 12	2	0.010016	19/01/21
5	1	0.60	02/12/20				
6	1	0.80	02/12/20				
7	1	1.00	02/12/20				
8	1	1.25	02/12/20				
9	1	1.50	02/12/20				
2	2	0.20	19/01/21				
3	2	0.40	19/01/21				
4	2	1.75	19/01/21				
5	2	2.00	19/01/21				
6	2	2.25	19/01/21				
7	2	2.50	19/01/21				
8	2	2.75	19/01/21				
9	2	3.00	19/01/21				
10	2	3.25	19/01/21				

a Total chloride molality in the solutions containing 0.04 mol kg^–1^ Tris buffer.

b The molalities of pure aqueous HCl in the cells used to determine standard potentials.

The Harned cells at PTB are routinely used for the certification of buffer solutions of ionic strengths up to 0.1 mol kg^–1^, and in one of our recent studies^9^ it was found that the high chloride molalities of the measurement solutions presented some difficulties. The apparent cause was the increased solubility of AgCl in solutions containing high concentrations of chloride ions.^12,13^ Gradual degradation of the reference electrodes due to the dissolution of the electrodeposited layer of silver chloride eventually results in irreversible damage, and Maksimov et al.^9^ found it necessary to measure solutions in a relatively short space of time in order to minimize this problem.

In this study, after measuring the first set of solutions the Ag_(s)_/AgCl_(s)_ electrodes that had been used in the highest molality solutions appeared brighter, and their potential against a Ag_(s)_/AgCl_(s)_ master electrode was found to have reduced, by an amount that depended on the chloride molality of the measurement solution. The effect occurred at lower molalities too. The fall in potential was about 0.1 mV after use of the electrode with the solution with a chloride molality of 0.2 mol kg^–1^, but around 0.25 mV after use in measurement solutions containing 1.0–1.5 mol kg^–1^ chloride. Before measuring the second set of solutions the electrodes were stored in ∼0.005 M HCl. During that time the potentials of the electrodes recovered significantly, increasing by up to 0.080 mV depending on the solution previously measured. The recovery time was however not sufficient to reach the original potential before the measurement of the first set of solutions. After measuring the second set of solutions the Ag_(s)_/AgCl_(s)_ electrode potentials dropped further, by around 0.140 mV. No obvious dependence on chloride molality could be observed here. However, looking at the total potential change from the time before any contact with the measurement solutions to the time after measuring the second set of solutions a dependence on the chloride molality is clearer. The total change ranged from around −0.15 mV for the remeasured 0.2 mol kg^–1^ solution to around −0.32 mV for the 3.25 mol kg^–1^ solution. We list in Table S2 the Ag_(s)_/AgCl_(s)_ electrodes used in this study, and the aqueous solutions they had previously been exposed to.

In section 1.3 of the Supporting Information we examine the consistency of measured potentials *within* each run by comparing values measured at 25 °C at the start, middle, and end of each sequence. We find that the values of the first measurements are generally less than those from the middle of the run by about 0.025 or 0.1 mV, while those at the end are greater by about 0.075 mV. No significant dependence upon solution composition was observed.

The quantity of interest in this measurement program is (*E* ^0^ – *E*), so it is important to consider to what extent the above changes in potential are compensated for in the determinations of the standard potentials, *E* ^0^. The experimental design, which was successful in previous measurements of dilute pH buffers, essentially assumes that the behavior of all Ag_(s)_/AgCl_(s)_ reference electrodes is the same and that their potentials are not affected by their history of past use. When used in solutions containing high chloride molalities this appears not to be the case, but it is not possible to quantify the effects exactly. Comparisons with other measurements, which are shown in Section 4, suggest that the offsets in (*E* ^0^ – *E*) may be of the order of a few tenths of a millivolt but are almost invariant with temperature.

The only way to avoid the difficulties described above appears to be the preparation of fresh reference electrodes for every measurement run, which was standard practice in some laboratories in the past. Another possible factor is a Harned cell design that can lead to enhanced migration of dissolved Ag^+^ to the half-cell containing the H_2_ electrode (Waters, Pers. comm.), but this does not affect the present measurements.

## 3 Treatment of the Data

The measured cell potentials, *E*_meas_, at the ambient H_2_ partial pressure in the cell are corrected to *p*H_2_ equal to 1 atm using the following relationship:^11^

$$E\left(pH_{2},1\;atm\right)=E_{meas}-RT/\left(2F\right)\cdot \ln\left(pH_{2}\right)$$ 2a

where

$$pH_{2}=P-pH_{2}O-pHCl+0.4\rho \cdot h\cdot g\cdot C$$ 2b

and *P* (atm) is atmospheric pressure at the time of the measurement, *p*H_2_O (atm) and *p*HCl (atm) are the equilibrium partial pressures of water and of HCl, respectively, above the solution at the temperature of the measurement. The final term in eq 4 is a further correction in which 0.4 is an empirical factor,^14^ ρ (g cm^–3^) is the density of the solution, *h* (mm) is the depth of immersion of the H_2_ electrode (here 70 mm), *g* (9.81 m s^–2^) is the gravitational constant, and C (1/101325 atm Pa^–1^) is a conversion factor from Pa to atm. The contribution of *p*HCl can be neglected. The values of *p*H_2_O are equal to *a*H_2_O·*p* ^o^(H_2_O), neglecting the small difference between partial pressure and fugacity, where *a*H_2_O is the water activity of the solution and *p* ^o^(H_2_O) (atm) the vapor pressure of pure water at the temperature of the measurement. Estimation of *p*HCl, *a*H_2_O, and ρ and their associated uncertainties is summarized in the Supporting Information to this work and that of Maksimov et al.^9^

The measured potentials of the buffer solutions, after adjustment to 1 atm *p*H_2_, were further adjusted as described by Maksimov et al.^8^ to be consistent with the standard potentials of Bates and Bower^15^ (column 7 of their Table 1) for ease of comparability. It is these adjusted potentials, *E*(adj.), that are tabulated in this work.

### 3.1 Standard Potentials

Standard potentials, *E* ^0^, of Cell A at each temperature were obtained from the measurements of 0.01 mol kg^–1^ HCl solutions, adjusted to 1 atm *p*H_2_, together with mean activity coefficients of HCl of Bates and Robinson.^16^ The effects of the very small deviations of the solution compositions from exactly 0.01 mol kg^–1^ were compensated for by adjusting the potentials *E* as described in Section 3.1 of Maksimov et al.^8^ and in the Supporting Information to this work. The temperatures at which some of cell potentials were determined, most notably 5, 10, and 15 °C, deviated from the exact integer temperatures by amounts that exceeded the uncertainty of the temperature measurement (±0.006 K). For this reason we have calculated *E* ^0^ for the experimental temperatures as measured (given to three decimal places) instead of rounded values, using a simple procedure described in the Supporting Information. However, the effect is small: for example the value of *E* ^0^ at a measured 10.018 °C differs from that at 10 °C by only −0.01 mV which is less than the uncertainty in the measurement of a cell potential.

All values of *E* ^0^, for both series of measurements, are shown in Figure 2a as deviations from the values of Bates and Bower^15^ (their equation 4). The individual electrodes behave similarly, although the potentials are slightly offset from one another by amounts that vary only slightly with temperature.

**Figure 2:**
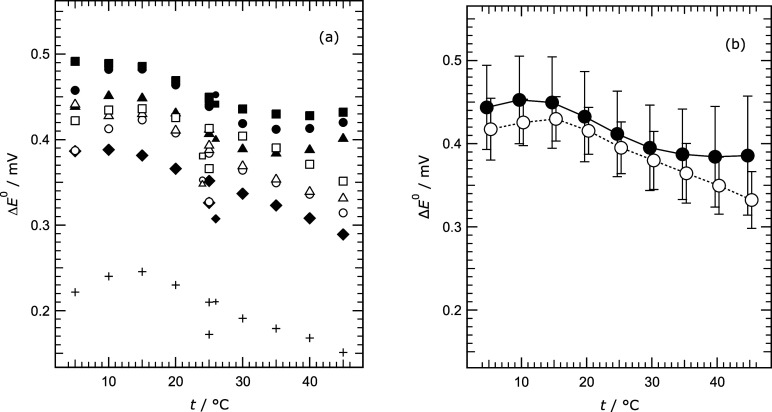
Differences between standard potentials determined in this work and those given by equation 4 of Bates and Bower^15^ (Δ*E* ^0^), for different cells. (a) Results for individual cells. The solid symbols and plus are series (1) results, and the open symbols are those of series (2). The values for the initial 25 °C measurements in each cell are offset horizontally and are reduced in size for ease of recognition. Symbols (series 1): plus – cell 1; dot – cell 2; solid triangle – cell 10; solid square – cell 11; solid diamond – cell 12. Symbols (series 2): circle – cell 1; triangle – cell 11; square – cell 12. (b) Mean values for each group, from Table 3. Symbols: dot – series (1), excluding the results for cell 1; circle – series 2.

We attribute the differences of about 0.3 to 0.5 mV from the results of Bates and Bower^15^ to electrode preparation and particularly the effects of electrode use over time as noted earlier. The results in Figure 2a for cell 1 deviate from the others by about 0.2 mV for reasons that are unknown, and they were discarded.

The mean values of Δ*E* ^0^ at each temperature were calculated separately for each series of measurements, and are shown in Figure 2b. The standard uncertainties shown on the plot for the series (1) results are chiefly associated with the scatter in the data, with about 20% accounted for by an assumed uncertainty in γ_HCl_ very close to the 0.0005 suggested by Bates et al.^17^ For the series (2) results (which are less scattered) the contribution of γ_HCl_ is in many cases the largest. For both series the additional contributions to the estimated uncertainty from that of the molality of the 0.01 mol kg^–1^ HCl and its temperature measurement are very small. Values of *E* ^0^ for both series are listed in Table 3.

**Table 3:** Standard Potentials (*E* ^0^), and Their Uncertainties (*u*) Determined in This Work

Series 1			Series 2		
*t* (°C)^a^	*E* ^0^ (V)	*u*(*E* ^0^) (mV)	*t* (°C)^a^	*E* ^0^ (V)	*u*(*E* ^0^) (mV)
5.014	0.234513	0.051	5.013	0.234487	0.037
10.018	0.231840	0.053	10.017	0.231813	0.028
15.014	0.228997	0.055	15.014	0.228977	0.027
20.008	0.225983	0.054	20.007	0.225966	0.028
25.003	0.222813	0.052	25.003	0.222796	0.031
30.005	0.219492	0.051	30.004	0.219477	0.035
35.003	0.216039	0.054	35.003	0.216016	0.036
40.005	0.212448	0.061	40.004	0.212413	0.034
45.009	0.208723	0.072	45.009	0.208670	0.034

a The calculation of *E* ^0^ was carried out at the listed measurement temperatures rather than rounded values. The uncertainty of the temperature measurements is ± 0.006 K.

### 3.2 Uncertainties

The standard uncertainties in the potentials *E* of the measurement solutions are about 0.026 mV for the first series of solutions, and a higher 0.056 mV for the second one. In contrast, the uncertainties in *E* ^0^ for the second series of solutions are the lower of the two, largely because the potentials for the different cells agree more closely (see Figure 2a). The overall estimated uncertainties in (*E* ^0^ – *E*) average just over 0.06 mV for both series.

## 4 Results and Discussion

Measured cell potentials, corrected to *p*H_2_ equal to 1 atm and adjusted to be consistent with the standard potentials of Bates and Bower^15^ are given in Table 4 together with values of the derived acidity function *Q* described below. Table S4 of the Supporting Information lists the same information together with the original measured potentials (*E*_meas_), the adjusted values *E*, and the other information required by eq 3.

**Table 4:** Harned Cell Results for 0.04 Mol kg^–1^ Tris Buffer in Aqueous NaCl at Ionic Strengths of 0.2 to 3.25 mol kg^–1^, Including Values of the Acidity Function^a^

Cell	*t* (°C)	*m*Cl^–^ (mol kg^–1^)	*m*NaCl (mol kg^–1^)	*m*TrisHCl (mol kg^–1^)	*m*Tris (mol kg^–1^)	*E*(adj.) (V)	*u*(*E*) (mV)	Acidity Function *Q* ^b^	*u*(*Q*)
3	25	0.20	0.160003	0.039972	0.040012	0.75859	0.026	–19.2597	0.0023
3	5	0.20	0.160003	0.039972	0.040012	0.76742	0.026	–20.6407	0.0024
3	10	0.20	0.160003	0.039972	0.040012	0.76533	0.026	–20.2720	0.0024
3	15	0.20	0.160003	0.039972	0.040012	0.76315	0.026	–19.9193	0.0025
3	20	0.20	0.160003	0.039972	0.040012	0.76092	0.026	–19.5827	0.0024
3	25	0.20	0.160003	0.039972	0.040012	0.75861	0.026	–19.2605	0.0023
3	30	0.20	0.160003	0.039972	0.040012	0.75622	0.026	–18.9511	0.0022
3	35	0.20	0.160003	0.039972	0.040012	0.75376	0.026	–18.6545	0.0023
3	40	0.20	0.160003	0.039972	0.040012	0.75121	0.026	–18.3694	0.0024
3	45	0.20	0.160003	0.039972	0.040012	0.74858	0.026	–18.0951	0.0028
3	25	0.20	0.160003	0.039972	0.040012	0.75864	0.026	–19.2618	0.0023
4	25	0.40	0.360084	0.039990	0.040014	0.74452	0.026	–19.4055	0.0023
4	5	0.40	0.360084	0.039990	0.040014	0.75433	0.026	–20.7883	0.0024
4	10	0.40	0.360084	0.039990	0.040014	0.75199	0.026	–20.4187	0.0024
4	15	0.40	0.360084	0.039990	0.040014	0.74956	0.026	–20.0656	0.0025
4	20	0.40	0.360084	0.039990	0.040014	0.74709	0.026	–19.7288	0.0024
4	25	0.40	0.360084	0.039990	0.040014	0.74455	0.026	–19.4067	0.0023
4	30	0.40	0.360084	0.039990	0.040014	0.74195	0.026	–19.0982	0.0022
4	35	0.40	0.360084	0.039990	0.040014	0.73927	0.026	–18.8025	0.0023
4	40	0.40	0.360084	0.039990	0.040014	0.73652	0.026	–18.5185	0.0024
4	45	0.40	0.360084	0.039990	0.040014	0.73369	0.026	–18.2455	0.0028
4	25	0.40	0.360084	0.039990	0.040014	0.74462	0.026	–19.4096	0.0023
5	25	0.60	0.559976	0.040002	0.040012	0.73642	0.026	–19.4956	0.0023
5	5	0.60	0.559976	0.040002	0.040012	0.74686	0.026	–20.8817	0.0024
5	10	0.60	0.559976	0.040002	0.040012	0.74435	0.026	–20.5108	0.0024
5	15	0.60	0.559976	0.040002	0.040012	0.74176	0.026	–20.1567	0.0025
5	20	0.60	0.559976	0.040002	0.040012	0.73913	0.026	–19.8190	0.0024
5	25	0.60	0.559976	0.040002	0.040012	0.73645	0.026	–19.4965	0.0023
5	30	0.60	0.559976	0.040002	0.040012	0.73370	0.026	–19.1876	0.0022
5	35	0.60	0.559976	0.040002	0.040012	0.73088	0.026	–18.8918	0.0023
5	40	0.60	0.559976	0.040002	0.040012	0.72799	0.026	–18.6078	0.0024
5	45	0.60	0.559976	0.040002	0.040012	0.72503	0.026	–18.3350	0.0028
5	25	0.60	0.559976	0.040002	0.040012	0.73652	0.026	–19.4993	0.0023
6	25	0.80	0.760116	0.039995	0.039990	0.73053	0.026	–19.5540	0.0023
6	5	0.80	0.760116	0.039995	0.039990	0.74142	0.026	–20.9430	0.0024
6	10	0.80	0.760116	0.039995	0.039990	0.73880	0.026	–20.5712	0.0024
6	15	0.80	0.760116	0.039995	0.039990	0.73609	0.026	–20.2162	0.0025
6	20	0.80	0.760116	0.039995	0.039990	0.73335	0.026	–19.8781	0.0024
6	25	0.80	0.760116	0.039995	0.039990	0.73056	0.026	–19.5554	0.0023
6	30	0.80	0.760116	0.039995	0.039990	0.72771	0.026	–19.2464	0.0022
6	35	0.80	0.760116	0.039995	0.039990	0.72480	0.026	–18.9508	0.0023
6	40	0.80	0.760116	0.039995	0.039990	0.72182	0.026	–18.6671	0.0024
6	45	0.80	0.760116	0.039995	0.039990	0.71878	0.026	–18.3947	0.0028
6	25	0.80	0.760116	0.039995	0.039990	0.73065	0.026	–19.5588	0.0023
7	25	1.00	0.959909	0.039980	0.040092	0.72598	0.026	–19.5997	0.0023
7	5	1.00	0.959909	0.039980	0.040092	0.73725	0.026	–20.9915	0.0024
7	10	1.00	0.959909	0.039980	0.040092	0.73452	0.026	–20.6186	0.0024
7	15	1.00	0.959909	0.039980	0.040092	0.73172	0.026	–20.2628	0.0025
7	20	1.00	0.959909	0.039980	0.040092	0.72888	0.026	–19.9238	0.0024
7	25	1.00	0.959909	0.039980	0.040092	0.72600	0.026	–19.6006	0.0023
7	30	1.00	0.959909	0.039980	0.040092	0.72306	0.026	–19.2912	0.0022
7	35	1.00	0.959909	0.039980	0.040092	0.72006	0.026	–18.9950	0.0023
7	40	1.00	0.959909	0.039980	0.040092	0.71700	0.026	–18.7111	0.0024
7	45	1.00	0.959909	0.039980	0.040092	0.71387	0.026	–18.4387	0.0028
7	25	1.00	0.959909	0.039980	0.040092	0.72607	0.026	–19.6033	0.0023
8	25	1.25	1.209971	0.040027	0.039913	0.72112	0.026	–19.6338	0.0023
8	5	1.25	1.209971	0.040027	0.039913	0.73280	0.026	–21.0293	0.0024
8	10	1.25	1.209971	0.040027	0.039913	0.72996	0.026	–20.6553	0.0024
8	15	1.25	1.209971	0.040027	0.039913	0.72706	0.026	–20.2984	0.0025
8	20	1.25	1.209971	0.040027	0.039913	0.72412	0.026	–19.9588	0.0024
8	25	1.25	1.209971	0.040027	0.039913	0.72114	0.026	–19.6349	0.0023
8	30	1.25	1.209971	0.040027	0.039913	0.71811	0.026	–19.3251	0.0022
8	35	1.25	1.209971	0.040027	0.039913	0.71503	0.026	–19.0288	0.0023
8	40	1.25	1.209971	0.040027	0.039913	0.71188	0.026	–18.7446	0.0024
8	45	1.25	1.209971	0.040027	0.039913	0.70867	0.026	–18.4721	0.0028
8	25	1.25	1.209971	0.040027	0.039913	0.72121	0.026	–19.6377	0.0023
9	25	1.50	1.460035	0.039991	0.039999	0.71724	0.026	–19.6650	0.0023
9	5	1.50	1.460035	0.039991	0.039999	0.72925	0.026	–21.0636	0.0024
9	10	1.50	1.460035	0.039991	0.039999	0.72631	0.026	–20.6881	0.0024
9	15	1.50	1.460035	0.039991	0.039999	0.72333	0.026	–20.3307	0.0025
9	20	1.50	1.460035	0.039991	0.039999	0.72031	0.026	–19.9904	0.0024
9	25	1.50	1.460035	0.039991	0.039999	0.71726	0.026	–19.6660	0.0023
9	30	1.50	1.460035	0.039991	0.039999	0.71415	0.026	–19.3557	0.0022
9	35	1.50	1.460035	0.039991	0.039999	0.71099	0.026	–19.0590	0.0023
9	40	1.50	1.460035	0.039991	0.039999	0.70777	0.026	–18.7747	0.0024
9	45	1.50	1.460035	0.039991	0.039999	0.70449	0.026	–18.5022	0.0028
9	25	1.50	1.460035	0.039991	0.039999	0.71732	0.026	–19.6686	0.0023
2	25	0.20	0.160255	0.039990	0.040003	0.75848	0.056	–19.2568	0.0025
2	5	0.20	0.160255	0.039990	0.040003	0.76736	0.056	–20.6397	0.0028
2	10	0.20	0.160255	0.039990	0.040003	0.76528	0.056	–20.2712	0.0025
2	15	0.20	0.160255	0.039990	0.040003	0.76310	0.056	–19.9184	0.0025
2	20	0.20	0.160255	0.039990	0.040003	0.76087	0.056	–19.5821	0.0025
2	25	0.20	0.160255	0.039990	0.040003	0.75857	0.056	–19.2602	0.0025
2	30	0.20	0.160255	0.039990	0.040003	0.75619	0.056	–18.9514	0.0025
2	35	0.20	0.160255	0.039990	0.040003	0.75374	0.056	–18.6554	0.0025
2	40	0.20	0.160255	0.039990	0.040003	0.75122	0.056	–18.3712	0.0024
2	45	0.20	0.160255	0.039990	0.040003	0.74861	0.056	–18.0978	0.0024
2	25	0.20	0.160255	0.039990	0.040003	0.75864	0.056	–19.2629	0.0025
3	25	0.40	0.360042	0.040003	0.039975	0.74445	0.056	–19.4026	0.0025
3	5	0.40	0.360042	0.040003	0.039975	0.75435	0.056	–20.7890	0.0028
3	10	0.40	0.360042	0.040003	0.039975	0.75200	0.056	–20.4190	0.0025
3	15	0.40	0.360042	0.040003	0.039975	0.74957	0.056	–20.0656	0.0025
3	20	0.40	0.360042	0.040003	0.039975	0.74710	0.056	–19.7291	0.0025
3	25	0.40	0.360042	0.040003	0.039975	0.74457	0.056	–19.4072	0.0025
3	30	0.40	0.360042	0.040003	0.039975	0.74196	0.056	–19.0987	0.0025
3	35	0.40	0.360042	0.040003	0.039975	0.73930	0.056	–18.8034	0.0025
3	40	0.40	0.360042	0.040003	0.039975	0.73656	0.056	–18.5200	0.0024
3	45	0.40	0.360042	0.040003	0.039975	0.73375	0.056	–18.2478	0.0024
3	25	0.40	0.360042	0.040003	0.039975	0.74467	0.056	–19.4111	0.0025
4	25	1.75	1.709978	0.040035	0.039958	0.71375	0.056	–19.6834	0.0025
4	5	1.75	1.709978	0.040035	0.039958	0.72615	0.056	–21.0883	0.0028
4	10	1.75	1.709978	0.040035	0.039958	0.72313	0.056	–20.7118	0.0025
4	15	1.75	1.709978	0.040035	0.039958	0.72006	0.056	–20.3530	0.0025
4	20	1.75	1.709978	0.040035	0.039958	0.71696	0.056	–20.0120	0.0025
4	25	1.75	1.709978	0.040035	0.039958	0.71384	0.056	–19.6871	0.0025
4	30	1.75	1.709978	0.040035	0.039958	0.71066	0.056	–19.3765	0.0025
4	35	1.75	1.709978	0.040035	0.039958	0.70745	0.056	–19.0798	0.0025
4	40	1.75	1.709978	0.040035	0.039958	0.70418	0.056	–18.7961	0.0024
4	45	1.75	1.709978	0.040035	0.039958	0.70087	0.056	–18.5242	0.0024
4	25	1.75	1.709978	0.040035	0.039958	0.71393	0.056	–19.6905	0.0025
5	25	2.00	1.959950	0.040008	0.040012	0.71069	0.056	–19.6981	0.0025
5	5	2.00	1.959950	0.040008	0.040012	0.72337	0.056	–21.1058	0.0028
5	10	2.00	1.959950	0.040008	0.040012	0.72028	0.056	–20.7284	0.0025
5	15	2.00	1.959950	0.040008	0.040012	0.71713	0.056	–20.3688	0.0025
5	20	2.00	1.959950	0.040008	0.040012	0.71398	0.056	–20.0274	0.0025
5	25	2.00	1.959950	0.040008	0.040012	0.71079	0.056	–19.7020	0.0025
5	30	2.00	1.959950	0.040008	0.040012	0.70756	0.056	–19.3910	0.0025
5	35	2.00	1.959950	0.040008	0.040012	0.70428	0.056	–19.0942	0.0025
5	40	2.00	1.959950	0.040008	0.040012	0.70096	0.056	–18.8101	0.0024
5	45	2.00	1.959950	0.040008	0.040012	0.69759	0.056	–18.5381	0.0024
5	25	2.00	1.959950	0.040008	0.040012	0.71088	0.056	–19.7053	0.0025
6	25	2.25	2.210013	0.040005	0.039994	0.70789	0.056	–19.7067	0.0025
6	5	2.25	2.210013	0.040005	0.039994	0.72080	0.056	–21.1165	0.0028
6	10	2.25	2.210013	0.040005	0.039994	0.71766	0.056	–20.7389	0.0025
6	15	2.25	2.210013	0.040005	0.039994	0.71445	0.056	–20.3785	0.0025
6	20	2.25	2.210013	0.040005	0.039994	0.71123	0.056	–20.0363	0.0025
6	25	2.25	2.210013	0.040005	0.039994	0.70799	0.056	–19.7106	0.0025
6	30	2.25	2.210013	0.040005	0.039994	0.70469	0.056	–19.3992	0.0025
6	35	2.25	2.210013	0.040005	0.039994	0.70136	0.056	–19.1020	0.0025
6	40	2.25	2.210013	0.040005	0.039994	0.69799	0.056	–18.8178	0.0024
6	45	2.25	2.210013	0.040005	0.039994	0.69457	0.056	–18.5457	0.0024
6	25	2.25	2.210013	0.040005	0.039994	0.70807	0.056	–19.7140	0.0025
7	25	2.50	2.460012	0.039997	0.040021	0.70535	0.056	–19.7133	0.0025
7	5	2.50	2.460012	0.039997	0.040021	0.71846	0.056	–21.1241	0.0028
7	10	2.50	2.460012	0.039997	0.040021	0.71525	0.056	–20.7457	0.0025
7	15	2.50	2.460012	0.039997	0.040021	0.71200	0.056	–20.3853	0.0025
7	20	2.50	2.460012	0.039997	0.040021	0.70873	0.056	–20.0429	0.0025
7	25	2.50	2.460012	0.039997	0.040021	0.70544	0.056	–19.7168	0.0025
7	30	2.50	2.460012	0.039997	0.040021	0.70210	0.056	–19.4054	0.0025
7	35	2.50	2.460012	0.039997	0.040021	0.69873	0.056	–19.1081	0.0025
7	40	2.50	2.460012	0.039997	0.040021	0.69531	0.056	–18.8238	0.0024
7	45	2.50	2.460012	0.039997	0.040021	0.69185	0.056	–18.5517	0.0024
7	25	2.50	2.460012	0.039997	0.040021	0.70552	0.056	–19.7199	0.0025
8	25	2.75	2.709906	0.039993	0.040082	0.70292	0.056	–19.7139	0.0025
8	5	2.75	2.709906	0.039993	0.040082	0.71610	0.056	–21.1211	0.0028
8	10	2.75	2.709906	0.039993	0.040082	0.71285	0.056	–20.7425	0.0025
8	15	2.75	2.709906	0.039993	0.040082	0.70956	0.056	–20.3821	0.0025
8	20	2.75	2.709906	0.039993	0.040082	0.70625	0.056	–20.0400	0.0025
8	25	2.75	2.709906	0.039993	0.040082	0.70292	0.056	–19.7141	0.0025
8	30	2.75	2.709906	0.039993	0.040082	0.69954	0.056	–19.4026	0.0025
8	35	2.75	2.709906	0.039993	0.040082	0.69613	0.056	–19.1056	0.0025
8	40	2.75	2.709906	0.039993	0.040082	0.69268	0.056	–18.8216	0.0024
8	45	2.75	2.709906	0.039993	0.040082	0.68918	0.056	–18.5500	0.0024
8	25	2.75	2.709906	0.039993	0.040082	0.70294	0.056	–19.7150	0.0025
9	25	3.00	2.960018	0.039999	0.040002	0.70063	0.056	–19.7120	0.0025
9	5	3.00	2.960018	0.039999	0.040002	0.71402	0.056	–21.1214	0.0028
9	10	3.00	2.960018	0.039999	0.040002	0.71072	0.056	–20.7422	0.0025
9	15	3.00	2.960018	0.039999	0.040002	0.70737	0.056	–20.3812	0.0025
9	20	3.00	2.960018	0.039999	0.040002	0.70401	0.056	–20.0384	0.0025
9	25	3.00	2.960018	0.039999	0.040002	0.70063	0.056	–19.7120	0.0025
9	30	3.00	2.960018	0.039999	0.040002	0.69720	0.056	–19.4000	0.0025
9	35	3.00	2.960018	0.039999	0.040002	0.69374	0.056	–19.1024	0.0025
9	40	3.00	2.960018	0.039999	0.040002	0.69024	0.056	–18.8182	0.0024
9	45	3.00	2.960018	0.039999	0.040002	0.68670	0.056	–18.5463	0.0024
9	25	3.00	2.960018	0.039999	0.040002	0.70062	0.056	–19.7118	0.0025
10	25	3.25	3.209959	0.040010	0.039998	0.69841	0.056	–19.7056	0.0025
10	5	3.25	3.209959	0.040010	0.039998	0.71198	0.056	–21.1164	0.0028
10	10	3.25	3.209959	0.040010	0.039998	0.70862	0.056	–20.7364	0.0025
10	15	3.25	3.209959	0.040010	0.039998	0.70523	0.056	–20.3750	0.0025
10	20	3.25	3.209959	0.040010	0.039998	0.70183	0.056	–20.0318	0.0025
10	25	3.25	3.209959	0.040010	0.039998	0.69840	0.056	–19.7051	0.0025
10	30	3.25	3.209959	0.040010	0.039998	0.69492	0.056	–19.3930	0.0025
10	35	3.25	3.209959	0.040010	0.039998	0.69142	0.056	–19.0954	0.0025
10	40	3.25	3.209959	0.040010	0.039998	0.68789	0.056	–18.8113	0.0024
10	45	3.25	3.209959	0.040010	0.039998	0.68432	0.056	–18.5395	0.0024
10	25	3.25	3.209959	0.040010	0.039998	0.69838	0.056	–19.7046	0.0025

a Column *m*Cl^–^ contains rounded values, and exact values can be calculated from the listed *m*NaCl and *m*TrisHCl. Prefix “*u*” in the column headers denotes an uncertainty. More complete results, including exact values of the rounded temperatures listed here, can be found in the Supporting Information.

b The acidity function *Q* is equal to ln­(*m*H^+^·γ_HCl_^2^), see eq 5.

In the buffer solutions the H^+^ molality is determined from the very slight dissociation of weak acid TrisH^+^ (to yield H^+^ and Tris), which is a function of the values of the thermodynamic equilibrium constant and activity coefficients of the three species. When considering the results of the measurements for these solutions it is therefore helpful to define the following acidity function, *Q*, which can be calculated directly from the measured cell potentials:

$$Q=\ln\left(mH^{+}\cdot {\gamma_{HCl}}^{2}\right)=\left(E^{0}-E\right)\cdot \left(F/RT\right)-\ln\left(mCl^{‐}\right)$$ 3

Note that this function is a natural logarithm rather than decadal, and is without a reversal of sign so that all calculated values are negative. By substituting for the product (*m*H^+^·γ_H_) in eq 5 it can be shown that

$$Q=\ln\left(K\left(\mathrm{Tris}H^{+}\right)\right)+\ln\left((m\mathrm{Tris}H^{+}/m\mathrm{Tris})\cdot {\gamma_{\mathrm{TrisHCl}}}^{2}/\gamma_{Tris}\right)$$ 4

where *K*(TrisH^+^) (mol kg^–1^) is the thermodynamic acid dissociation constant of TrisH^+^. The value of this dissociation constant is known,^10^ and calculations show that the molalities of TrisH^+^ and Tris at equilibrium will differ very little from their stoichiometric values (which are known from the preparation of the solutions). Consequently the cell potential differences (*E* ^0^ – *E*) are to a large extent a measure of the quantity γ_TrisHCl_^2^/γ_Tris_ in the solutions.

Values of the acidity function (on a log_10_ basis) at all temperatures are shown in Figure 3a, together with results from Maksimov et al.^9^ for the same solutions. The top axis indicates the practical salinities of artificial seawater corresponding to the ionic strengths on the bottom *x*-axis. The line on the plot represents values of the acidity function calculated for the Tris buffer in aqueous NaCl using the draft thermodynamic model of Clegg et al.^6^ for solutions at 25 °C. This includes parameters for the interaction of Tris with Na^+^ and a number of other ions, but not parameters for Na^+^-TrisH^+^-Cl^–^ or H^+^-TrisH^+^-Cl^–^ interactions (the latter do not influence the calculated acidity function for reasons explained by Maksimov et al.^9^).

**Figure 3:**
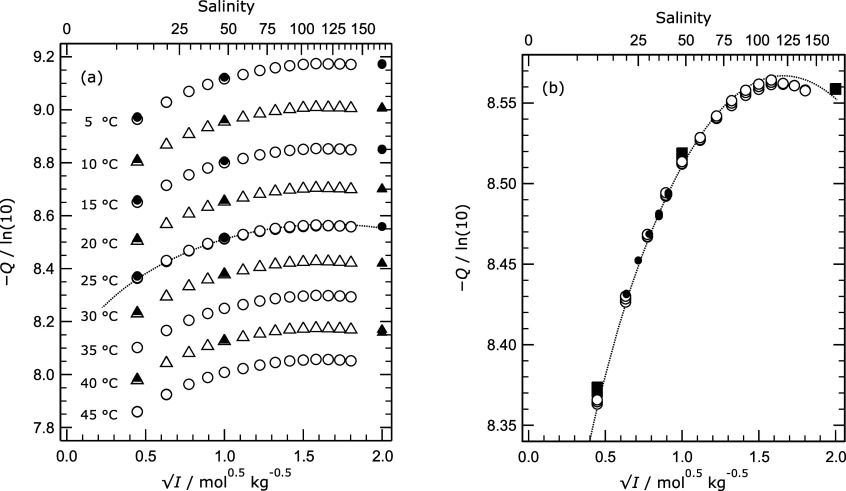
Values of the acidity function (eq 5, but on a decadal logarithmic basis) plotted against the square root of ionic strength (*I*). (a) Open symbols – all data from this study (Table 4), at the temperatures indicated on the graph; solid symbols – results of Maksimov et al.^9^ Dotted line – model calculation for 25 °C (see text). (b) Results for 25 °C only, with the addition of values in artificial seawater calculated from measured cell potentials of DelValls and Dickson^2^ (dots, for salinities 20 to 40).

Results for 25 °C are plotted in Figure 3b and include values determined from the Harned cell measurements of DelValls and Dickson^2^ of Tris buffer in artificial seawater. The results of Maksimov et al. are consistently high (in terms of −*Q*) relative to those of this study. Values of *Q* from DelValls and Dickson agree quite closely with the both other sets of data, which is expected because Na^+^ and Cl^–^ ions together make up about 90 mol % of the solute content of artificial seawater. At the highest ionic strengths the predictions of the draft model deviate from our results.

Values of the molality and activity coefficient quotient defined in eq 6 are plotted in Figure 4, to illustrate how ln­(γ_TrisHCl_^2^/γ_Tris_) varies with temperature at each ionic strength. At the lowest measured ionic strength it decreases monotonically with temperature, but at 1.75 mol kg^–1^ and above there is a maximum value at about 15 to 20 °C.

**Figure 4:**
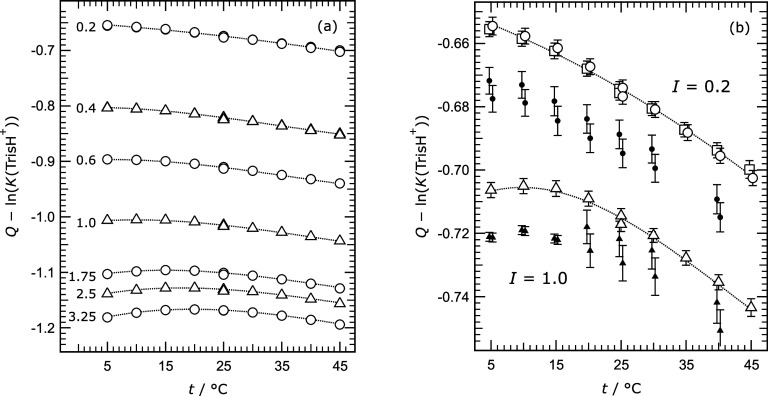
Values of the acidity function with the influence of the thermodynamic equilibrium constant *K*(TrisH^+^) removed (eq 6) plotted against temperature (*t*). (a) Symbols – measurements from this study at all ionic strengths as indicated at the left-hand side of the plot. Values for ionic strength 3.25 mol kg^–1^ are offset vertically by −0.05 for clarity. The dotted lines are for guidance only. (b) Results from this study at ionic strength 0.2 and 1.0 mol kg^–1^ (same data as in (a)) compared with results of Maksimov et al.^9^ for the same solution compositions. Symbols: squares – this study (set 1); circles – this study (set 2); triangles – this study (set 1); dots – Maksimov et al.^9^ (cells 73 and 74); small solid triangles – Maksimov et al.^9^ (cells 75 and 76). All values for ionic strength 1.0 mol kg^–1^ are offset vertically by +0.3 in order to increase the resolution of the plot, and horizontal offsets of ± 0.25 °C are applied so that error bars can be distinguished. The dotted lines through the data from this study are for guidance only.

Figure 4b compares our results at two ionic strengths with those of Maksimov et al.^9^ for the same solutions. There is a consistent offset between the two data sets of about 0.007 in the *y*-axis variable, which is equivalent to 0.18 mV in (*E* ^0^ – *E*) at 25 °C. This exceeds the estimated standard uncertainties of most of the measurements. At present, it seems likely that the results presented in this work are more nearly correct, in part because our data agree more closely with the draft Pitzer model predictions especially at the lowest ionic strength of 0.2 mol kg^–1^ for which the model is expected to be most accurate.

The trends in temperature of both data sets in Figure 4b agree well, which suggests that if accurate values of (*E* ^0^ – *E*) can be established at one temperature (most likely 25 °C) then the data for other temperatures, at the same composition, can be adjusted. Errors in the value of the thermodynamic dissociation constant of TrisH^+^ in eq 6 affect the derived quantity ln­(γ_TrisHCl_^2^/γ_Tris_) by almost the same amount at each temperature, irrespective of the total chloride molality of the solution. (The effect on ln­(*m*TrisH^+^/*m*Tris) is very small.) Bates and Hetzer^10^ state an uncertainty in the measurement of ln­(*K*(TrisH^+^)) of ±0.0014, although the value obtained from their fitted equation differs from that in their Table 2 by −0.0063 on a natural log basis.

### 4.1 Modeling

In the Pitzer model,^7^ the excess Gibbs energy of solutions and derived quantities such as the solute activity coefficients are calculated as summations of the effects of interactions between pairs and triplets of solute species. Each interaction is characterized by one or more parameters whose values – determined by fitting to thermodynamic data – vary with both temperature and pressure. The parameters are explained in section 2 of Clegg et al.,^6^ and also defined in their glossary of symbols. The calculation of the cell potentials using a Pitzer model has been discussed by Maksimov et al.,^9^ who also presented a table of the relevant binary and ternary interaction parameters. Clegg et al.^6^ developed a preliminary model, for 25 °C only, of solutions containing Tris buffer in artificial seawater. It is parameters from that model that are used here. They are listed, together with their values, in Table 5 and discussed in more detail below.

**Table 5:** Pitzer Model Parameters for 25 °C Used to Calculate Cell Potentials

Ions c and a		β^(0)^_c,a_	β^(1)^_c,a_	*C* ^(0)^_c,a_	Note	Source
Na^+^	Cl^–^	0.0753591	0.277031	0.000703969	^a^, ^b^	^18^
TrisH^+^	Cl^–^	0.0426783	0.196255	–0.00144509	^b^	^6,22^
						
Species n		λ_n,n_	μ_n,n,n_		Note	Source
Tris		–0.0051635	0.000703		^c^	^22^
						
Species n and i		λ_n,i_	ζ_n,i,Cl_		Note	Source
Tris	Na^+^	0.02632	0		^d^	^6,22^
Tris	TrisH^+^	–0.01241	0		^e^	^22^
						
Cations i and j		θ_i,j_	ψ_i,j,Cl_		Note	Source
Na^+^	TrisH^+^	0	0		^d^	see text

a These parameters from Møller^18^ were adopted by Humphreys et al.^25^ and Clegg et al.^6^ for their models of artificial seawater and Tris buffer in artificial seawater.

b The model coefficient α, which is used with parameter β^(1)^_c,a_, is equal to 2.0 for both salts.

c The parameters have a negligible influence on the model calculated cell potential and are omitted from eq 8 for *A*_mix_.

d Clegg et al.^6^ note in their Table 2 that the value −0.02632 for this parameter is actually that for (θ_Na,TrisH_ – λ_Tris,Na_), and for the purpose of calculating the cell potential of the buffer a value of zero is currently assigned to θ_Na,TrisH_ and 0.02632 assigned to λ_Tris,Na_. The source of this value is Table 10 of Lodeiro et al.^22^

e The terms containing this parameter cancel for the Pitzer model expression for the cell potential of the equimolal buffer and therefore do not appear in eq 8.

In Figure 5 we examine some of the results shown in Figure 3b, which compares values of *Q* determined from cell potentials from different sources with model calculations. The influences that make Δ*Q* in the figure greater or smaller can be characterized as the following sum of terms:

$$\Delta Q\equiv \left\{meas.error\right\}+\Delta \ln(K\left(TrisH^{+})\right)+\Delta \left\{cation-anion\;\gamma \;terms\right\}+\Delta A_{mix}$$ 5

where the first term on the right represents measurement error, the difference between the measured (*E* ^0^ – *E*) in eq 5 and the true value; Δln­(*K*(TrisH^+^)) is the difference between the true value of ln­(*K*(TrisH^+^)) and that used to calculate *Q* from eq 6; Δ­{cation–anion γ terms} is the error associated with the cation–anion interactions TrisH^+^-Cl^–^ and Na^+^-Cl^–^ in the model; and Δ*A*_mix_ is the error associated with the mixture terms in the model, some of which were set to zero in the calculations of *Q* shown in the figures. Quantity *A*_mix_, the contribution of Pitzer model mixture terms to ln­(γ_TrisHCl_^2^/γ_Tris_) in eq 6, is given by

$$A_{\mathrm{mix}}=2mNa^{+}\cdot \left[\left(\theta_{Na,\mathrm{TrisH}}-\lambda_{\mathrm{Tris},Na}\right)+mB\cdot \left(\psi_{Na,\mathrm{TrisH},Cl}+\zeta_{\mathrm{Tris},Na,Cl}\right)\right]+m{Na}^{+}\cdot m{Cl}^{‐}\cdot \left(\psi_{Na,\mathrm{TrisH},Cl}-\zeta_{\mathrm{Tris},Na,Cl}\right)$$ 6

where *m*B is the buffer molality of 0.04 mol kg^–1^. Contributions from the self-interaction of Tris are negligible and are omitted from the expression, and those for Tris–TrisH^+^ interactions cancel.

**Figure 5:**
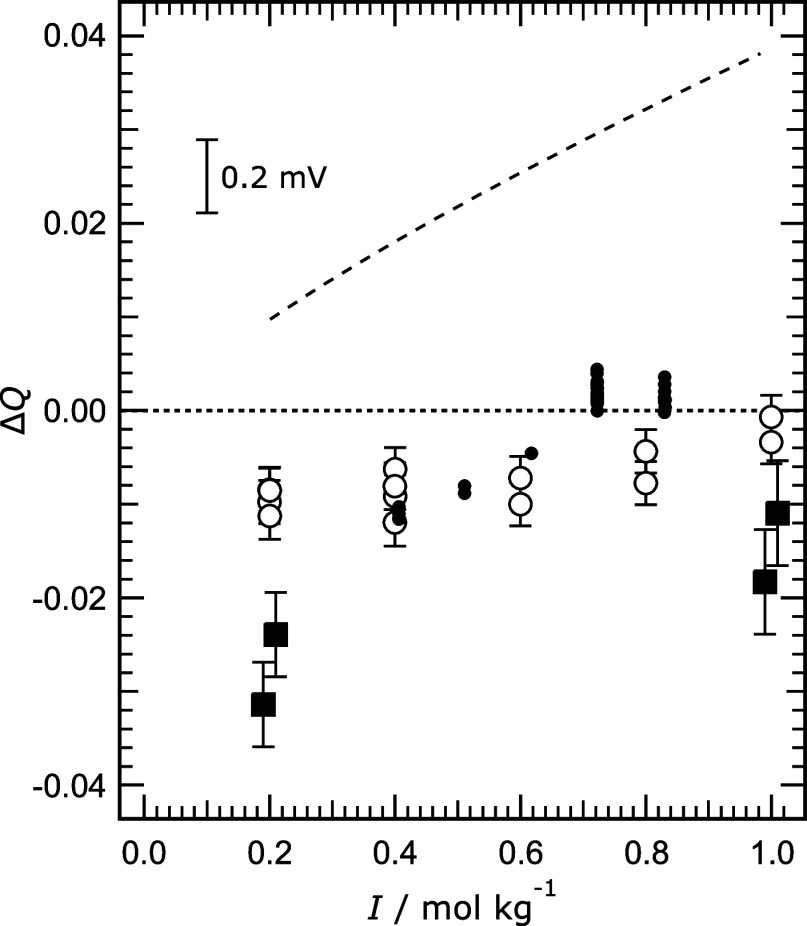
Differences between values of *Q* (eq 5) from various sources at 25 °C and those calculated using the Pitzer model for 0.04 mol kg^–1^ Tris buffer in aqueous NaCl, plotted against ionic strength (*I*). Symbols: large circles – from cell potentials determined in this work; solid squares – from cell potentials determined by Maksimov et al.;^9^ small dots – from cell potentials of DelValls and Dickson^2^ for Tris buffer in artificial seawater. Dashed line: calculated using the model of Clegg et al.^6^ for Tris buffer in artificial seawater. At upper left we show the change in *Q* for a change in cell potential of 0.2 mV.

The molality of H^+^ in the solutions is very low and also eq 6 does not include the activity coefficient of H^+^, thus implying that interactions involving H^+^ have no influence on the calculated value of *Q*. This is confirmed by sensitivity calculations carried out by Clegg et al.^6^ for the cell potential of 0.04 mol kg^–1^ Tris buffer in salinity 35 artificial seawater. They show, in their Figure 2, the individual contributions of uncertainties in all Pitzer model parameters and equilibrium constants to the total estimated uncertainty. The major contributors in terms of percentages of the total variance are as follows: TrisH^+^-Cl^–^ parameters, 73%; Na^+^-Cl^–^ parameters, 17%; and ln­(*K*(TrisH^+^)), 5%. There are no contributions from H^+^ interactions with other species above the 0.0014% level.

The comparison of measurements and model calculations yields Δ*Q* less than zero (Figure 5), by an amount corresponding, at the lowest ionic strengths, to about −0.3 mV in (*E* ^0^ – *E*). There are several possible reasons for this negative value:

**1**. The measured (*E* ^0^ – *E*) are too small. As explained in Section 2.2, the solutions with high chloride molalities were found to affect some of the electrodes and *reduce* measured cell potentials *E* which would tend to yield (*E* ^0^ – *E*) that are too high rather than too low (unless compensated for by a corresponding reduction in *E* ^0^). This term therefore does not seem likely to account for the negative Δ*Q* in the figure.
**2**. Values of ln­(*K*(TrisH^+^)) used to calculate *Q* are too large. This quantity was obtained from equation 3 of Bates and Hetzer^10^ which yields −18.58614 (after conversion to a natural logarithm). However, the measured value in their Table 2 is equivalent to −18.59245. If this is assumed to be more nearly correct then the difference between the two would account for −0.0063 of the negative Δ*Q* shown on the plot.
**3**. The cation–anion interactions in the model are poorly predicted. Those for Na^+^-Cl^–^, from Møller,^18^ were used in the model of Waters and Millero^19^ but have not yet been compared with the critical review of Archer^20^ (an alternative source of parameters). Furthermore, parameters for TrisH^+^-Cl^–^ interactions determined from a fit to a single set of isopiestic measurements^21^ do not appear to be fully consistent with the cell potentials and derived ln­(*K*(TrisH^+^)) of Bates and Hetzer,^10^ (see Figures 4 and 5 of Clegg et al.^6^). Calculated values of *Q* are very sensitive to the TrisH^+^-Cl^–^ interactions in the model and Clegg et al. showed that a refitting of the data to achieve greater overall consistency could yield a change in the calculated potential in an artificial seawater matrix of 0.5 mV or so. A careful reassessment of the Harned cell data for aqueous TrisHCl-Tris solutions used to obtain *K*(TrisH^+^), and the osmotic coefficient data for aqueous TrisHCl, is recommended. We note that further osmotic coefficient measurements are currently being made (Miladinovic, Pers. comm.).
**4**. The quantity *A*_mix_ is not fully characterized, because some parameters are not yet known accurately and were set to zero. The only nonzero parameter in our calculations of *Q* is the combination (θ_Na,TrisH_ – λ_Tris,Na_), from Table 10 of Lodeiro et al.^22^ (see also the notes to our Table 5). In order to account for the negative value of Δ*Q* in Figure 5 the calculated *A*_mix_ term defined in eq 8 would have to be too large. The study of Lodeiro et al. suggests that ζ_Tris,Na,Cl_ is negative, but there is contradictory information concerning ψ_Na,TrisH,Cl_: values presented by Lodeiro et al. are negative (−0.0094 and −0.001455) whereas recent isopiestic measurements suggest a small positive value of about 0.003 (Miladinovic, Pers. comm.). Until the values of the parameters are better established it is unclear what the net effect on *A*_mix_ will be.

Similar considerations to those discussed above apply to the data of Maksimov et al.^9^ in Figure 5. The more negative values of Δ*Q* relative to those of our values for the same solutions are assumed to be the result of an offset in (*E* ^0^ – *E*) attributable to the condition of their electrodes.

We have included in Figure 5 a dashed line indicating the calculated difference between the value of *Q* for Tris buffer in artificial seawater and that for the buffer in aqueous NaCl of the same ionic strength. This illustrates the influence of the other solutes in seawater. The difference between this line, and the values of Δ*Q* from the measurements of DelValls and Dickson^2^ is equivalent to about 0.8 mV in (*E* ^0^ – *E*), as is also shown in Figure 3 of Clegg et al.^6^ who discuss the reasons for the differences.

Stoichiometric dissociation constants of TrisH^+^ (K*­(TrisH^+^)) in NaCl solutions have been determined by Millero et al.^23^ (by titration) and by Palmer and Wesolowski^24^ (using a concentration cell with hydrogen electrodes). They are defined by the following expression:

$$K^{*}\left(\mathrm{Tris}H^{+}\right)=mH^{+}\cdot m\mathrm{Tris}/m\mathrm{Tris}H^{+}=K\left(\mathrm{Tris}H^{+}\right)\cdot \gamma_{\mathrm{Tris}H}/\left(\gamma_{H}\cdot \gamma_{\mathrm{Tris}}\right)$$ 7

where *K*(TrisH^+^) is the thermodynamic dissociation constant at the temperature of interest. The results of the two studies can be employed to test the model used for the comparisons in Figure 5, although it is important to note that there are necessary assumptions involved in the determination of K*­(TrisH^+^) from both types of measurement. Modeled and calculated pK*­(TrisH^+^) at 25 °C are shown in Figure 6 and agree very well, and suggest that the prediction of the activity coefficients in eq 9 (and in eq 6 for *Q*) is quite accurate. The small differences shown in the inset for the lower molality range are most likely due to the assumptions noted above and are not discussed in this work.

**Figure 6:**
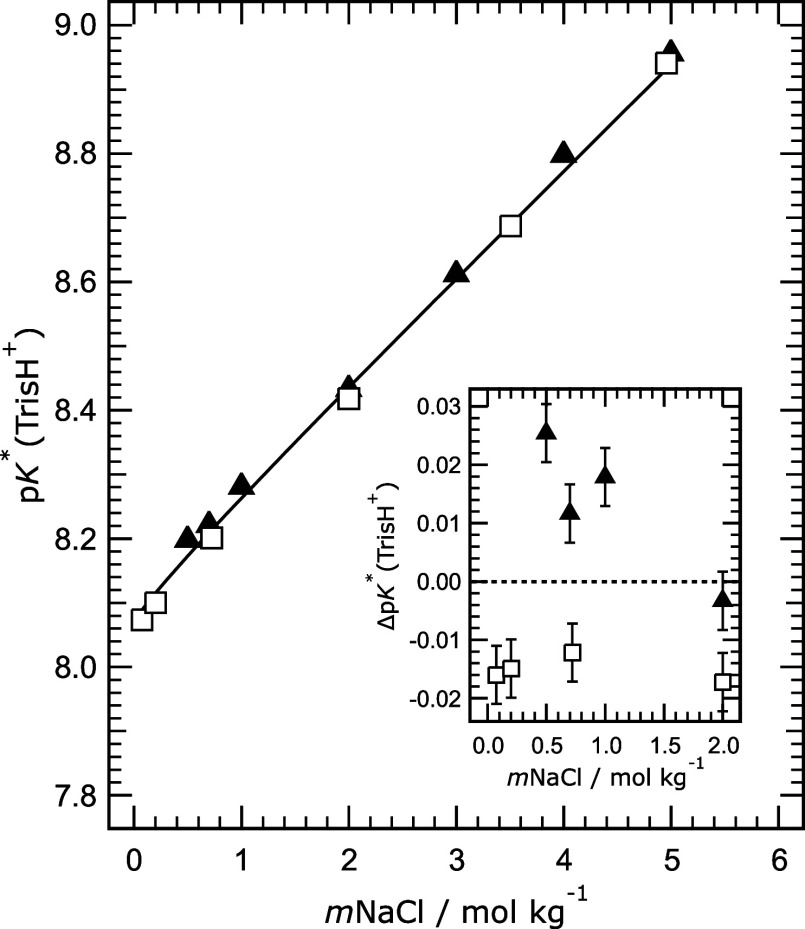
Comparison of measured and calculated pK*­(TrisH^+^) in aqueous NaCl solutions at 25 °C, plotted against sodium chloride molality (*m*NaCl). Symbols: solid triangle – titration measurements of Millero et al.;^23^ square – from cell potentials of Palmer and Wesolowski.^24^ Line – calculated using the model of Clegg et al.^6^ Inset: difference between measured and calculated values (showing experimental uncertainties) up to 2.0 mol kg^–1^ chloride molality. Note: the data of Millero et al.^23^ include a value for 6.0 mol kg^–1^ which is not shown here.

In conclusion, the refinement of the model to yield more accurate values of the cation–anion and *A*_mix_ contributions to the model-calculated value of *Q* can be expected to yield better agreement with the data. The sensitivity tests of Clegg et al.,^6^ referred to earlier, suggest that improvements to the TrisH^+^-Cl^–^ parameters are likely to have a large effect and our comparisons above show that the values for the thermodynamic equilibrium constant should also be reassessed.

## 5 Summary

We have measured cell potentials, and derived values of an acidity function *Q*, of solutions containing equimolal Tris buffer in NaCl aqueous solutions. The cell potentials at 0.2 mol kg^–1^ and 1.0 mol kg^–1^ ionic strength differ from the results of Maksimov et al.^9^ by about 0.2 – 0.4 mV, which can probably be attributed to some degradation of the reference electrodes in the solutions containing high molalities of chloride. Acidity functions calculated using a Pitzer model using available interaction parameters at 25 °C (some of which are unknown or poorly characterized) agree with the experimental values to within a closer 0.2 to 0.3 mV at the same ionic strengths noted above. A previous sensitivity study,^6^ and comparisons made in this work, suggest that much of this difference may be accounted for by the uncertainty in the available thermodynamic dissociation constant of TrisH^+^ and the Pitzer parameters for TrisH^+^-Cl^–^ interactions. These new measurements, when combined with the results of our previous studies^8,9^ and other literature data, should enable an accurate Pitzer ion interaction model of the buffer solutions to be developed. The interactions of the TrisH^+^ and Tris buffer species with the other major ions of seawater are being addressed in further studies using Harned cells, and by isopiestic measurements of osmotic coefficients. The results described here, for Tris buffer in aqueous NaCl, will be the key element of the larger thermodynamic model of Tris buffer in artificial seawater.

## Supplementary Material



## Notes

Description of electrode preparation; determination of standard potentials; densities and water activities of the measurement solutions; tabulation of original measured potentials; tabulation of derived acidity function values including uncertainties (PDF)
