# Supplementary material for: Activity Coefficients of HCl in Solutions Related to “Tris” Buffers in Artificial Seawater. III. Tris Buffer + NaCl + H2O, from 0.2 to 3.25 mol kg–1 Ionic Strength and from 5 to 45 °C
Source: J Chem Eng Data. 2025 Dec 5;71(1):33–45. doi: 10.1021/acs.jced.5c00450 (PMC12794158; doi:10.1021/acs.jced.5c00450)
Supplement: Supplementary file 1 [file je5c00450_si_001.pdf]

Supporting Information

Activity Coefficients of HCl in Solutions Related to 'Tris' Buffers in Artificial Seawater. III. Tris buffer + NaCl + H_2O, From 0.2 to 3.25 mol kg^{-1} Ionic Strength and From 5 °C to 45 °C

Frank Bastkowski,*[,a] Beatrice Sander,[a] and Simon L. Clegg*[b]

[a] Physikalisch-Technische Bundesanstalt (PTB), Bundesallee 100, 38116 Braunschweig, Germany
[b] School of Environmental Sciences, University of East Anglia, Norwich NR4 7TJ, United Kingdom

* Corresponding authors. *E-mail*: frank.bastkowski@ptb.de, and s.clegg@uea.ac.uk

Contents

	Page
1. Use of the Harned cells in this study	2
1.1 The $Ag_{(s)}/AgCl_{(s)}$ (chloride) electrodes	2
1.2 The Pt hydrogen electrodes	3
1.3 Measurement procedure	3
2. Determination of standard potentials, E^0	5
3. Densities of the solutions	6
4. Estimation of the water activities of the solutions	6
5. Results	7
6. Tables	7
References	15

This Supporting Information (SI) describes, first of all, some of the particular difficulties experienced in the Harned cell measurements in this study. Next there is information that supports the description in the main text of the determination of standard potentials of the cells (E^0). We also describe the estimation of the densities of the solutions, their water activities, and equilibrium partial pressures of HCl which are needed in order to adjust cell potentials to a standard 1 atm pressure of H_2.

The complete experimental results are tabulated, including the measured potentials, ambient pressures, densities and water activities of the solutions, and the acidity function and its estimated uncertainty.

1. Use of the Harned cells in this study

A typical PTB Harned Cell is shown in Figure S1, below. The preparation of electrodes is described by Bates,[1] and the methods used at the National Metrology Laboratory of Japan are detailed in our previous studies.[2-3] The earlier of those studies[2] includes comments from Andrew G. Dickson of the Scripps Institution of Oceanography concerning the preparation of electrodes in his laboratory, where different. We summarise here the methods used at PTB.

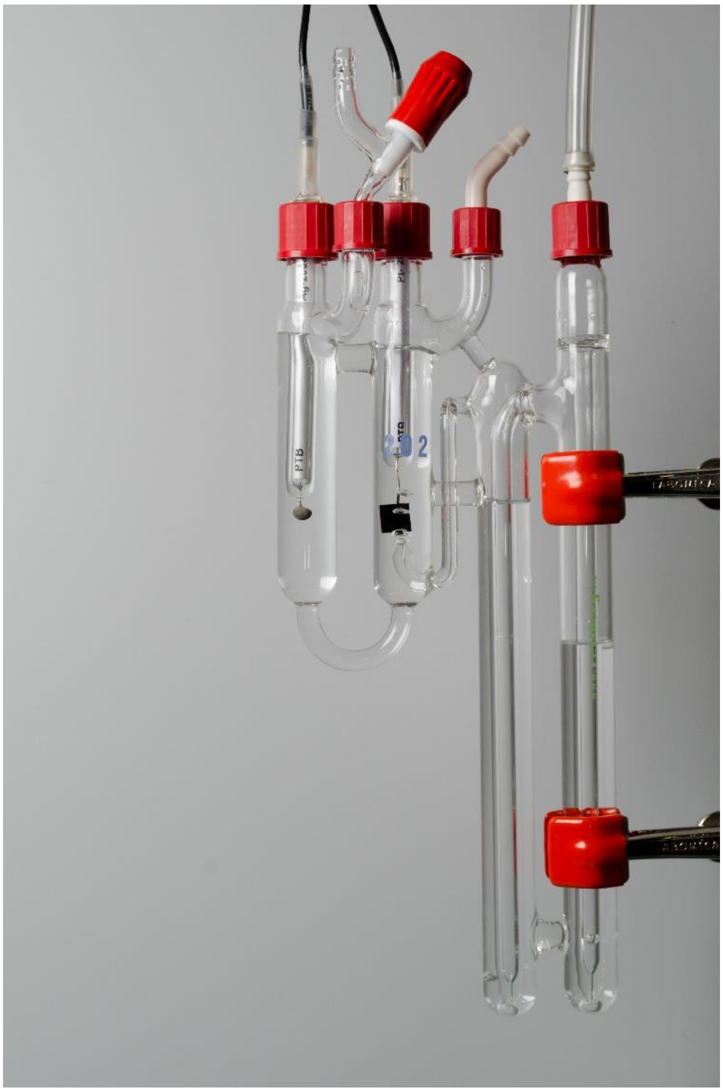

Figure S1. A Harned cell. Hydrogen gas enters the apparatus at the top right and passes through two pre-saturator bubbler tubes (the first one is shown held by the red clamps) that contain the same solution that is being measured. The gas stream then passes into the half-cell containing the Pt hydrogen electrode (the dark square shape) via a small tube just beneath the electrode. It bubbles up over the electrode and out of the half-cell via a vent. This part of the cell is joined to a second half-cell by a U-shaped capillary tube which contains the $Ag_{(s)}/AgCl_{(s)}$ chloride electrode (the irregular rounded shape).

1.1 The $Ag_{(s)}/AgCl_{(s)}$ (chloride) electrodes

The electrodes used in these measurements were prepared in 2008. They are formed on a Pt wire, with an eyelet at the end, and sealed in a glass body. The eyelet is first cleaned for 5 minutes in hot 6 M nitric acid, then rinsed with water and kept in boiling distilled water for 15 min. The wire is then dried and annealed in a methanol flame. To prepare the electrodes 10 to 12 thin layers of ≥99.5% pure Ag_2O (Merck company), thoroughly dispersed in ultrapure water, are applied to the eyelet. Each layer of Ag_2O dispersion is dried at room temperature for 30 min and then converted

to Ag by heating at 450 °C for 15 min. The number of applied Ag_2O layers is chosen so as to reach a final mass of applied silver of 100-150 mg. The resulting spherical silver electrode is then electrochemically chloridized in 1 M HCl with at DC current of 10 mA vs. a platinum cathode for 30 – 45 min depending on the actual mass of the silver. In the completed electrodes approximately 5 mm of the Pt wire is still uncovered and hence is exposed to the solution in the Harned cell.

The electrodes are stored in a 0.005 M aqueous HCl, and this solution is replaced every few months. Before use, the electrodes' potentials are compared against each other by differential potentiometry in a 0.01 M NaCl solution. Electrodes within a potential difference window of 50 μV are used for the measurements.

Prior to measurements performed for this work, the electrodes had most recently been used in the analysis of solutions containing 0.01 mol kg^{-1} HCl and 0.99 or 4.99 mol kg^{-1} NaCl, followed by storage for 16 months in 0.005 mol kg^{-1} HCl.

1.2 The Pt hydrogen electrodes

The electrode bodies used for the preparation of the Pt electrodes are the same as those used for the $Ag_{(s)}/AgCl_{(s)}$ electrodes (Pt wire sealed in a glass tube). A Pt plate (of area 1.8 cm^2) is forged to the free end of the Pt wire using a hammer, with both the wire and the plate heated by a natural gas burner. The raw Pt electrode is then treated for a few seconds in hot 50% aqua regia. After that a standard cleaning procedure is applied, as follows:

The electrode is first treated with hot (but not boiling) 6 M nitric acid for 5 min, then rinsed with deionized water and placed in boiling deionized water for 15 min. Following this the Pt plate is calcined with an ethanol burner and then electrochemically cleaned in 0.1 M nitric acid by applying 200 mA cm^{-2} for 1 min as anode vs. Pt, and 5 min as cathode vs. Pt. The electrodes are then placed in deionised water for 1 h.

After the above cleaning procedure the blank Pt electrodes are finally platinized with platinum black using the Pb-acetate method described by Bates.[1]

For the measurements performed in this study an existing set of Pt electrodes were replatinized before every measurement run. These electrodes were treated with hot 50% aqua regia so as to remove the existing Pt black layer, then rinsed with deionized water and cleaned using the standard procedure described above. The electrodes were then placed in deionized water for 1 h, and platinized again using the Pb-acetate method noted above.

Before use the Pt electrodes are stored in deionized water which is replaced at least every 2 months. In contrast to the $Ag_{(s)}/AgCl_{(s)}$ electrodes, the Pt electrodes were *not* checked for their equivalence by differential potentiometry before use.

1.3 Measurement procedure

The equipment used is listed in Table S1. Twelve cells were used in each measurement run. They are immersed in a temperature control bath, in which the level of the control liquid (water) is approximately that of the top of the measurement solution. The cells, with the electrodes already

mounted, were first filled with the measurement solution and then treated with argon for 1 h followed by hydrogen for 2 h before an initial 25 °C measurement was made. After completing this measurement, a series of further measurements were performed from 5 °C to 45 °C in 5 °C steps, and then finally a third one at 25 °C.

Data recording at each measurement temperature was started when the temperature was stable to better than 20 mK over a period of a minute. Recording was stopped when the voltage drift of the most recent 11 values, at each temperature, was less then 50 μV h^{-1}. The reported voltages, temperatures and pressures are averages of these 11 values. In each run the minimum number of cycles (consecutive potential measurements of the 12 cells) is 21 and the maximum number of cycles is 30. The temperature homogeneity in the bath was better than ± 0.015 °C and the stability was better than ± 0.010 °C.

No particular difficulties were encountered in the measurements relative to what is normally experienced with standard pH buffer solutions (the usual use of the Harned cells in the PTB laboratory). However, it was found that the first results for the two lowest molality solutions appeared low relative to our other data and for this reason these two solutions were remeasured. Both sets of results are presented.

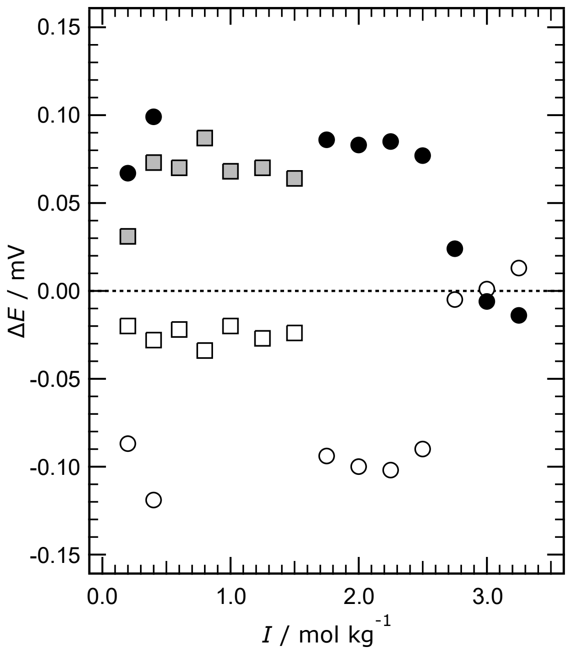

Figure S2. Differences of the first and last potential measurements at 25 °C from the middle value for each cell (ΔE) in each measurement run plotted against ionic strength (I). Symbols: open squares – (E[first] – E[mid.]) for the first group of measurements (carried out in December 2020); open circles – (E[first] – E[mid.]) for the second group of measurements (carried out in January 2021); shaded squares – (E[last] – E[mid.]) for first group of measurements; dots – (E[last] – E[mid.]) for second group of measurements.

The measurement of cell potentials at 25 °C three times in each run allows us to roughly test the consistency of the data. Figure S2 shows the differences between the first and third measurements relative to the middle value. The earlier group of measurements (made in 2020) show consistent behaviour: the first measurements are low by about 0.025 mV and the last ones high by about 0.075 mV. There is no apparent relationship with solution composition. In the case of the second group the cell potential measured first has a larger deviation, about -0.1 mV. The three highest ionic strength solutions measured show negligible offsets. The deviations in the figure compare with estimated standard uncertainties of ±0.026 mV (first group of measurements) and ±0.056 mV (second group). The reasons for the deviations shown in Figure S2 are unclear, but may possibly be

caused by some combination of insufficient time to equilibrate after changes in temperature and to the characteristics (past history of use) of individual electrodes.

The measurements were made in two separate runs, in December 2020 and January 2021. The identifiers of the $Ag_{(s)}/AgCl_{(s)}$ electrodes are listed in Table S2 together with the dates and aqueous solutions measured for their most recent uses prior to this study. It can be seen that the majority of the reference electrodes used in measurements in 2021 had also been used for the first run in 2020. Before 2020 some of those electrodes had been used in acidified NaCl solutions of 0.1, 1.0 and 5.0 mol kg^{-1} ionic strength.

2. Determination of standard potentials, E^0

The mean activity coefficients of HCl (γ_{HCl}) tabulated by Bates and Robinson[4] are for 0.01 *m* HCl and rounded Celsius temperatures. In order to base the calculation of the standard potentials of the cells upon these values the following adjustments were carried out to the Harned cell measurements of the dilute aqueous HCl solutions.

(i) The measured potentials of the cells containing the dilute HCl of molality *m* were adjusted to values for 0.01 mol kg^{-1} HCl exactly (at the temperature of the measurement) using the following expression:

$$E(0.01) = E + 2RT/F \cdot [\ln(m \cdot \gamma_{HCl}) - \ln(0.01 \cdot \gamma_{HCl(0.01)})] \qquad \text{(S1)}$$

where E is the measured potential (after correction to 1 atm pH_2), and $E(0.01)$ is the adjusted value. The mean activity coefficients γ_{HCl} (for molality *m* and temperature *T*), and $\gamma_{HCl(0.01)}$ (for molality 0.01 mol kg^{-1} and temperature *T*) are both calculated using the model of the thermodynamic properties of HCl of Holmes et al.[5] with parameters given in column "BP, Model I" of their Table 3.

(ii) The listed γ_{HCl} of Bates and Robinson[4] for 0.01 mol kg^{-1} HCl at the rounded temperature is adjusted to an estimate for the exact temperature *T* of the measurement using this expression:

$$\gamma_{HCl}(T) = \gamma_{HCl}(T_{rnd}, \text{Bates}) \cdot [\gamma_{HCl}(T, \text{Holmes}) \ / \ \gamma_{HCl}(T_{rnd}, \text{Holmes})] \qquad \text{(S2)}$$

where T and T_{rnd} indicate values for measurement temperature and the rounded temperature, respectively. The mean activity coefficients in the square brackets in eq S2 are calculated using the model of Holmes et al.,[5] as indicated.

Finally, the standard cell potential E^0 at the exact measurement temperature T is calculated from:

$$E^0 = E(0.01) + 2RT/F \cdot \ln(0.01 \cdot \gamma_{HCl}(T)) \qquad \text{(S3)}$$

It should be noted that the adjustments made for these molality and temperature differences are both very small, and less than the uncertainties in the E^0 values themselves. (Of the two, the adjustment for molality is the larger.) The uncertainties are estimated using normal methods of uncertainty propagation (e.g., JCGM[6]). The standard uncertainties in the temperature, HCl molality and atmospheric pressure were 0.0058 K (thermometer certificate), 1.75×10^{-6} mol kg^{-1} (coulometric titration), and 3.5 – 5 Pa (barometer), respectively.

The largest contributions to the uncertainties listed in Table 3 are those associated with the averaging of the values obtained from the different cells, and our assumed uncertainty of the mean activity coefficients of Bates and Robinson (±0.00045) which is very close to the ±0.0005 suggested for 25 °C by Bates et al.[7] The group using Harned cells at the National Metrology Institute of Japan assume an uncertainty of ±0.0003 in γ_{HCl} at all temperatures, based upon this value and the assumption that it is an extended uncertainty. (This value of ±0.0003 is used in our other studies.[2-3]) All of these seem reasonable values when compared with a modern assessment of γ_{HCl} in pure aqueous HCl at 25 °C.[8]

3. Densities of the solutions

The densities of the solutions, at each experimental temperature, are used in the final term in eq 2b for the adjustment of the measured potentials to pH_2 equal to 1 atm. The influence on quantities obtained from the value of $(E - E^0)$ is that of the difference between the JetCorr terms for the test solution and for the 0.01 *m* HCl. This will be a difference of the order of only a few µV.

In the same way as described by Maksimov et al.,[3] densities of the solutions were estimated by assuming additivity of apparent molar volumes of the solutes NaCl, TrisHCl, and Tris in the solution:

$$V_{Tot.} = m\text{NaCl}\cdot V^{\Phi}(\text{NaCl}) + m\text{TrisHCl}\cdot V^{\Phi}(\text{TrisHCl}) + m\text{Tris}\cdot V^{\Phi}(\text{Tris}) + 1000/\rho(\text{Water}) \quad \text{(S4)}$$

where $V_{Tot.}$ is the estimated total volume of a solution containing 1000 g H_2O, V^{Φ} ($cm^3\ mol^{-1}$) is the apparent molar volume of the indicated solute at its molality (m) in the mixture, and ρ(Water) is the density of pure water in g cm^{-3}. The density of the solution mixture is obtained from the total volume as given above, and the total mass (based upon the same 1000 g of water solvent).

Densities of pure water, and apparent molar volumes of aqueous HCl and aqueous TrisHCl, were obtained as described in the Supporting Information to Maksimov et al.[2] Values for aqueous NaCl were obtained from Clegg and Wexler,[9] and Tris from the following equation fitted to the data of Ford et al.:[10]

$$V^{\Phi}(\text{Tris}) = 69.26 + 0.0754\cdot T \quad \text{(S5)}$$

Uncertainties in the densities of the solutions make only a very small contribution to those of the adjustments of the measured potentials to a pH_2 of 1 atm. The solutions containing Tris buffer in aqueous NaCl are either dominated by NaCl (the solutions at 1.0 and 4.0 mol kg^{-1} ionic strength), or are so dilute that any errors in the estimated densities will be small. Consequently, for these solutions we have followed the usual PTB practise of assigning an uncertainty of $\pm 1\times 10^{-4}$ g cm^{-3} to both the HCl and buffer solutions.

4. Estimation of the water activities of the solutions

The approach used in this work is very similar to that for the solutions described in Supporting Information to the studies of Maksimov et al.[2-3] The water activities of the solutions, at each experimental temperature, are used in the pH_2O term in eq 2b for the adjustment of cell potentials to exactly 1 atm pH_2 and are calculated using the Pitzer model. We use $TrisH^+$-Cl^- parameters from Table 2 of Tishchenko,[11] those for H^+-Cl^- from Holmes et al.,[5] and those for Na^+-Cl^- from Archer.[12] The parameter for Tris-Na^+ interactions ($\lambda_{Tris,Na}$, invariant with temperature) was taken from Millero et al.,[13] which is similar in value to the estimates from other data discussed by Lodeiro et al.[14] We

also use mixture parameters for H^+-TrisH^+-Cl^- interactions (known at 25 °C only) from Bates and Macaskill,[15] and those for H^+-Na^+-Cl^- (also known at 25 °C only) from Harvie and Weare.[16] For these buffer solutions, Pitzer parameters for H^+ interactions will have a negligible influence on water activity, but they were included in our calculations for completeness. Water activities at 5, 25, and 40 °C for some representative solutions are listed in Table S3. The water activities of the dilute aqueous HCl solutions have also been calculated, and are very close to unity: 0.9996 to 0.9997 depending on temperature.

The contributions of the pH_2O term to the adjusted values of the cell potentials are largest at the highest measured temperatures, as is illustrated for HCl-TrisHCl solutions in section 4 of the Supporting Information to Maksimov et al.[2] As can be seen in Table S3 here, the water activities of the Tris buffer solutions are predicted to vary little with temperature, and water activities calculated for 25 °C (for which Pitzer parameters are generally more accurately known) could have been used at all temperatures without much loss of accuracy.

Our previous experience[2-3] has shown that uncertainties of the water activities of the measurement solutions have only a small influence on the estimated uncertainty of a mean activity coefficient or acidity constant calculated from the cell potentials. Furthermore, the Tris buffer solutions consist mainly of aqueous NaCl for which the water activities are accurately known. For these reasons we have not estimated uncertainties in the water activities and have assumed them to be zero.

5. Results

Complete experimental results are listed in Table S4. This contains the pressure, P, for each measurement; cell potentials including the original measured values (before the correction to pH_2 equal to 1 atm); the estimated uncertainties of the acidity function obtained from the measurements; and the water activity and density of each solution (used in the pressure correction).

6. Tables

Table S1. Equipment used for measurements

Device	Manufacturer and model	Use
Harned cells (12 units)	Rettberg GmbH, custom-built	Potential measurements
Thermostatic bath / circulator	Lauda Proline PV36	Temperature control
Through-flow cooler	Lauda DLK 45	Cooling the thermostatic bath
Thermometer	ASL-WIKA CTR5000	Measuring temperature
Temperature probes	PT100 (4 units), PTB self-built	Temperature sensing in the bath
Multimeter	Keysight, model 3458A	Measuring Harned cell potentials
Pressure meter	Setra 370 (Setra Systems Inc.)	Measuring atmospheric pressure

Table S2. Identifiers of the $Ag_{(s)}/AgCl_{(s)}$ electrodes and their previous use [a]

Run	Date [b]	*m*Cl$^-$ [c] (mol kg^{-1})	Electrode	Previous use of electrode	Date of previous use
1	02/12/20	0.01	PH2_AG498	0.01 *m*HCl	13/08/19
1	02/12/20	0.01	PH2_AG499	(unknown)	>10 years ago
1	02/12/20	0.20	PH2_AG501	0.01 *m*HCl	13/08/19
1	02/12/20	0.40	PH2_AG502	0.01 *m*HCl + 0.09 *m*NaCl	13/08/19
1	02/12/20	0.60	PH2_AG503	(unknown)	>10 years ago
1	02/12/20	0.80	PH2_AG505	0.01 *m*HCl + 0.09 *m*NaCl	13/08/19
1	02/12/20	1.00	PH2_AG506	0.01 *m*HCl + 0.09 *m*NaCl	13/08/19
1	02/12/20	1.25	PH2_AG508	0.01 *m*HCl + 0.99 *m*NaCl	13/08/19
1	02/12/20	1.50	PH2_AG509	0.01 *m*HCl + 0.99 *m*NaCl	13/08/19
1	02/12/20	0.01	PH2_AG510	0.01 *m*HCl + 0.99 *m*NaCl	13/08/19
1	02/12/20	0.01	PH2_AG511	0.01 *m*HCl + 0.99 *m*NaCl	13/08/19
1	02/12/20	0.01	PH2_AG512	0.01 *m*HCl + 4.99 *m*NaCl	07/08/18
2	19/01/21	0.01	PH2_AG497	0.01 *m*HCl	13/08/19
2	19/01/21	0.20	PH2_AG498	0.01 *m*HCl (Run 1)	02/12/20
2	19/01/21	0.40	PH2_AG500	0.01 *m*HCl	13/08/19
2	19/01/21	1.75	PH2_AG501	*m*Cl = 0.2 mol kg^{-1} (Run 1)	02/12/20
2	19/01/21	2.00	PH2_AG502	*m*Cl = 0.4 mol kg^{-1} (Run 1)	02/12/20
2	19/01/21	2.25	PH2_AG503	*m*Cl = 0.6 mol kg^{-1} (Run 1)	02/12/20
2	19/01/21	2.50	PH2_AG505	*m*Cl = 0.8 mol kg^{-1} (Run 1)	02/12/20
2	19/01/21	2.75	PH2_AG506	*m*Cl = 1.0 mol kg^{-1} (Run 1)	02/12/20
2	19/01/21	3.00	PH2_AG508	*m*Cl = 1.25 mol kg^{-1} (Run 1)	02/12/20
2	19/01/21	3.25	PH2_AG509	*m*Cl = 1.50 mol kg^{-1} (Run 1)	02/12/20
2	19/01/21	0.01	PH2_AG512	0.01 *m*HCl (Run 1)	02/12/20
2	19/01/21	0.01	PH2_AG514	0.01 *m*HCl + 4.99 *m*NaCl	07/08/18

[a] All electrodes were made at PTB in 2008. [b] The dates on which the measurements described in this work were carried out. [c] Chloride molalities of the solutions measured in this study (values of 0.01 are 0.01 *m* HCl solutions).

Table S3. Calculated water activities of some solutions containing Tris buffer in aqueous NaCl

*m*Cl$^-$ (mol kg^{-1})	*a*H_2O (5 °C)	*a*H_2O (25 °C)	*a*H_2O (40 °C)
0.2	0.9927	0.9927	0.9927
1.0	0.9666	0.9961	0.9660
3.25	0.8850	0.8822	0.8812

.

Table S4: Harned Cell Results For 0.04 mol kg^{-1} Tris Buffer in Aqueous NaCl Solutions at Ionic Strengths of 0.2 to 3.25 mol kg^{-1}, Including Values of the Acidity Function (eq 3) [a]

Cell	t (°C)	P (atm)	mCl$^-$ (mol kg^{-1})	mNaCl (mol kg^{-1})	mTrisHCl (mol kg^{-1})	mTris (mol kg^{-1})	E(meas.)^a (V)	$E^{\circ a}$ (V)	E(adj.)^a (V)	u(E) (mV)	Acidity function Q^b	u(Q)	a_{H_2O}	ρ (g cm^{-3})
3	25.004	0.992787	0.20	0.160003	0.039972	0.040012	0.758539	0.759004	0.758592	0.026	-19.2597	0.0023	0.9927	1.0067
3	5.014	0.991162	0.20	0.160003	0.039972	0.040012	0.767683	0.767860	0.767417	0.026	-20.6407	0.0024	0.9927	1.0102
3	10.018	0.990090	0.20	0.160003	0.039972	0.040012	0.765546	0.765783	0.765330	0.026	-20.2720	0.0024	0.9927	1.0098
3	15.014	0.988920	0.20	0.160003	0.039972	0.040012	0.763287	0.763602	0.763152	0.026	-19.9193	0.0025	0.9927	1.0090
3	20.008	0.987401	0.20	0.160003	0.039972	0.040012	0.760930	0.761351	0.760919	0.026	-19.5827	0.0024	0.9927	1.0080
3	25.003	0.985673	0.20	0.160003	0.039972	0.040012	0.758464	0.759024	0.758612	0.026	-19.2605	0.0023	0.9927	1.0067
3	30.005	0.984451	0.20	0.160003	0.039972	0.040012	0.755885	0.756616	0.756221	0.026	-18.9511	0.0022	0.9927	1.0052
3	35.003	0.983560	0.20	0.160003	0.039972	0.040012	0.753195	0.754143	0.753755	0.026	-18.6545	0.0023	0.9927	1.0036
3	40.005	0.982107	0.20	0.160003	0.039972	0.040012	0.750357	0.751593	0.751210	0.026	-18.3694	0.0024	0.9927	1.0017
3	45.009	0.980622	0.20	0.160003	0.039972	0.040012	0.747355	0.748964	0.748578	0.026	-18.0951	0.0028	0.9927	0.9996
3	25.002	0.978829	0.20	0.160003	0.039972	0.040012	0.758403	0.759055	0.758644	0.026	-19.2618	0.0023	0.9927	1.0067
4	25.004	0.992787	0.40	0.360084	0.039990	0.040014	0.744473	0.744935	0.744523	0.026	-19.4055	0.0023	0.9861	1.0147
4	5.014	0.991162	0.40	0.360084	0.039990	0.040014	0.754599	0.754775	0.754332	0.026	-20.7883	0.0024	0.9862	1.0186
4	10.018	0.990090	0.40	0.360084	0.039990	0.040014	0.752206	0.752442	0.751989	0.026	-20.4187	0.0024	0.9862	1.0180
4	15.014	0.988920	0.40	0.360084	0.039990	0.040014	0.749701	0.750015	0.749565	0.026	-20.0656	0.0025	0.9862	1.0172
4	20.008	0.987401	0.40	0.360084	0.039990	0.040014	0.747105	0.747524	0.747092	0.026	-19.7288	0.0024	0.9861	1.0160
4	25.003	0.985673	0.40	0.360084	0.039990	0.040014	0.744406	0.744963	0.744551	0.026	-19.4067	0.0023	0.9861	1.0147
4	30.005	0.984451	0.40	0.360084	0.039990	0.040014	0.741616	0.742343	0.741948	0.026	-19.0982	0.0022	0.9861	1.0131
4	35.003	0.983560	0.40	0.360084	0.039990	0.040014	0.738716	0.739658	0.739271	0.026	-18.8025	0.0023	0.9861	1.0113
4	40.005	0.982107	0.40	0.360084	0.039990	0.040014	0.735674	0.736903	0.736519	0.026	-18.5185	0.0024	0.9861	1.0094
4	45.009	0.980622	0.40	0.360084	0.039990	0.040014	0.732476	0.734075	0.733689	0.026	-18.2455	0.0028	0.9861	1.0073
4	25.002	0.978829	0.40	0.360084	0.039990	0.040014	0.744387	0.745036	0.744625	0.026	-19.4096	0.0023	0.9861	1.0147
5	25.004	0.992787	0.60	0.559976	0.040002	0.040012	0.736377	0.736836	0.736424	0.026	-19.4956	0.0023	0.9795	1.0224
5	5.014	0.991162	0.60	0.559976	0.040002	0.040012	0.747124	0.747299	0.746856	0.026	-20.8817	0.0024	0.9797	1.0268
5	10.018	0.990090	0.60	0.559976	0.040002	0.040012	0.744566	0.744800	0.744347	0.026	-20.5108	0.0024	0.9797	1.0261
5	15.014	0.988920	0.60	0.559976	0.040002	0.040012	0.741902	0.742214	0.741764	0.026	-20.1567	0.0025	0.9796	1.0251
5	20.008	0.987401	0.60	0.559976	0.040002	0.040012	0.739147	0.739564	0.739131	0.026	-19.8190	0.0024	0.9796	1.0239
5	25.003	0.985673	0.60	0.559976	0.040002	0.040012	0.736304	0.736858	0.736446	0.026	-19.4965	0.0023	0.9795	1.0224
5	30.005	0.984451	0.60	0.559976	0.040002	0.040012	0.733370	0.734093	0.733698	0.026	-19.1876	0.0022	0.9795	1.0208
5	35.003	0.983560	0.60	0.559976	0.040002	0.040012	0.730332	0.731268	0.730881	0.026	-18.8918	0.0023	0.9795	1.0190

5	40.005	0.982107	0.60	0.559976	0.040002	0.040012	0.727156	0.728377	0.727994	0.026	-18.6078	0.0024	0.9795	1.0170
5	45.009	0.980622	0.60	0.559976	0.040002	0.040012	0.723828	0.725417	0.725031	0.026	-18.3350	0.0028	0.9795	1.0148
5	25.002	0.978829	0.60	0.559976	0.040002	0.040012	0.736282	0.736928	0.736516	0.026	-19.4993	0.0023	0.9795	1.0224
6	25.004	0.992787	0.80	0.760116	0.039995	0.039990	0.730485	0.730941	0.730529	0.026	-19.5540	0.0023	0.9729	1.0301
6	5.014	0.991162	0.80	0.760116	0.039995	0.039990	0.741694	0.741868	0.741425	0.026	-20.9430	0.0024	0.9732	1.0349
6	10.018	0.990090	0.80	0.760116	0.039995	0.039990	0.739017	0.739250	0.738797	0.026	-20.5712	0.0024	0.9731	1.0340
6	15.014	0.988920	0.80	0.760116	0.039995	0.039990	0.736233	0.736543	0.736093	0.026	-20.2162	0.0025	0.9730	1.0329
6	20.008	0.987401	0.80	0.760116	0.039995	0.039990	0.733371	0.733785	0.733353	0.026	-19.8781	0.0024	0.9729	1.0316
6	25.003	0.985673	0.80	0.760116	0.039995	0.039990	0.730424	0.730975	0.730563	0.026	-19.5554	0.0023	0.9729	1.0301
6	30.005	0.984451	0.80	0.760116	0.039995	0.039990	0.727390	0.728109	0.727713	0.026	-19.2464	0.0022	0.9728	1.0284
6	35.003	0.983560	0.80	0.760116	0.039995	0.039990	0.724259	0.725190	0.724803	0.026	-18.9508	0.0023	0.9728	1.0265
6	40.005	0.982107	0.80	0.760116	0.039995	0.039990	0.720994	0.722208	0.721824	0.026	-18.6671	0.0024	0.9728	1.0244
6	45.009	0.980622	0.80	0.760116	0.039995	0.039990	0.717584	0.719163	0.718777	0.026	-18.3947	0.0028	0.9728	1.0222
6	25.002	0.978829	0.80	0.760116	0.039995	0.039990	0.730419	0.731062	0.730650	0.026	-19.5588	0.0023	0.9729	1.0301
7	25.004	0.992787	1.00	0.959909	0.039980	0.040092	0.725937	0.726390	0.725978	0.026	-19.5997	0.0023	0.9661	1.0376
7	5.014	0.991162	1.00	0.959909	0.039980	0.040092	0.737516	0.737689	0.737246	0.026	-20.9915	0.0024	0.9666	1.0428
7	10.018	0.990090	1.00	0.959909	0.039980	0.040092	0.734737	0.734969	0.734516	0.026	-20.6186	0.0024	0.9665	1.0418
7	15.014	0.988920	1.00	0.959909	0.039980	0.040092	0.731858	0.732166	0.731717	0.026	-20.2628	0.0025	0.9663	1.0406
7	20.008	0.987401	1.00	0.959909	0.039980	0.040092	0.728896	0.729308	0.728876	0.026	-19.9238	0.0024	0.9662	1.0392
7	25.003	0.985673	1.00	0.959909	0.039980	0.040092	0.725862	0.726410	0.725998	0.026	-19.6006	0.0023	0.9661	1.0376
7	30.005	0.984451	1.00	0.959909	0.039980	0.040092	0.722741	0.723456	0.723060	0.026	-19.2912	0.0022	0.9661	1.0358
7	35.003	0.983560	1.00	0.959909	0.039980	0.040092	0.719522	0.720447	0.720060	0.026	-18.9950	0.0023	0.9660	1.0339
7	40.005	0.982107	1.00	0.959909	0.039980	0.040092	0.716176	0.717382	0.716999	0.026	-18.7111	0.0024	0.9660	1.0318
7	45.009	0.980622	1.00	0.959909	0.039980	0.040092	0.712687	0.714255	0.713870	0.026	-18.4387	0.0028	0.9660	1.0295
7	25.002	0.978829	1.00	0.959909	0.039980	0.040092	0.725838	0.726478	0.726066	0.026	-19.6033	0.0023	0.9661	1.0376
8	25.004	0.992787	1.25	1.209971	0.040027	0.039913	0.721080	0.721529	0.721117	0.026	-19.6338	0.0023	0.9576	1.0468
8	5.014	0.991162	1.25	1.209971	0.040027	0.039913	0.733072	0.733244	0.732801	0.026	-21.0293	0.0024	0.9583	1.0525
8	10.018	0.990090	1.25	1.209971	0.040027	0.039913	0.730185	0.730415	0.729962	0.026	-20.6553	0.0024	0.9581	1.0514
8	15.014	0.988920	1.25	1.209971	0.040027	0.039913	0.727201	0.727507	0.727058	0.026	-20.2984	0.0025	0.9579	1.0500
8	20.008	0.987401	1.25	1.209971	0.040027	0.039913	0.724143	0.724552	0.724120	0.026	-19.9588	0.0024	0.9577	1.0485
8	25.003	0.985673	1.25	1.209971	0.040027	0.039913	0.721012	0.721556	0.721144	0.026	-19.6349	0.0023	0.9576	1.0468
8	30.005	0.984451	1.25	1.209971	0.040027	0.039913	0.717800	0.718509	0.718114	0.026	-19.3251	0.0022	0.9575	1.0449
8	35.003	0.983560	1.25	1.209971	0.040027	0.039913	0.714496	0.715414	0.715027	0.026	-19.0288	0.0023	0.9574	1.0429
8	40.005	0.982107	1.25	1.209971	0.040027	0.039913	0.711064	0.712261	0.711877	0.026	-18.7446	0.0024	0.9573	1.0408
8	45.009	0.980622	1.25	1.209971	0.040027	0.039913	0.707495	0.709050	0.708665	0.026	-18.4721	0.0028	0.9573	1.0385

8	25.002	0.978829	1.25	1.209971	0.040027	0.039913	0.720990	0.721626	0.721214	0.026	-19.6377	0.0023	0.9576	1.0468
9	25.004	0.992787	1.50	1.460035	0.039991	0.039999	0.717202	0.717647	0.717235	0.026	-19.6650	0.0023	0.9488	1.0558
9	5.014	0.991162	1.50	1.460035	0.039991	0.039999	0.729523	0.729694	0.729251	0.026	-21.0636	0.0024	0.9498	1.0620
9	10.018	0.990090	1.50	1.460035	0.039991	0.039999	0.726539	0.726768	0.726315	0.026	-20.6881	0.0024	0.9495	1.0607
9	15.014	0.988920	1.50	1.460035	0.039991	0.039999	0.723477	0.723781	0.723331	0.026	-20.3307	0.0025	0.9492	1.0592
9	20.008	0.987401	1.50	1.460035	0.039991	0.039999	0.720339	0.720745	0.720313	0.026	-19.9904	0.0024	0.9490	1.0576
9	25.003	0.985673	1.50	1.460035	0.039991	0.039999	0.717131	0.717671	0.717259	0.026	-19.6660	0.0023	0.9488	1.0558
9	30.005	0.984451	1.50	1.460035	0.039991	0.039999	0.713842	0.714546	0.714151	0.026	-19.3557	0.0022	0.9487	1.0539
9	35.003	0.983560	1.50	1.460035	0.039991	0.039999	0.710465	0.711376	0.710988	0.026	-19.0590	0.0023	0.9485	1.0518
9	40.005	0.982107	1.50	1.460035	0.039991	0.039999	0.706966	0.708153	0.707769	0.026	-18.7747	0.0024	0.9485	1.0496
9	45.009	0.980622	1.50	1.460035	0.039991	0.039999	0.703336	0.704878	0.704493	0.026	-18.5022	0.0028	0.9484	1.0473
9	25.002	0.978829	1.50	1.460035	0.039991	0.039999	0.717103	0.717735	0.717323	0.026	-19.6686	0.0023	0.9488	1.0558
2	25.003	0.980244	0.20	0.160255	0.039990	0.040003	0.758244	0.758877	0.758482	0.056	-19.2568	0.0025	0.9927	1.0067
2	5.013	0.980994	0.20	0.160255	0.039990	0.040003	0.767474	0.767775	0.767358	0.056	-20.6397	0.0028	0.9927	1.0102
2	10.017	0.981107	0.20	0.160255	0.039990	0.040003	0.765353	0.765702	0.765276	0.056	-20.2712	0.0025	0.9927	1.0098
2	15.014	0.981990	0.20	0.160255	0.039990	0.040003	0.763122	0.763526	0.763096	0.056	-19.9184	0.0025	0.9927	1.0090
2	20.007	0.982157	0.20	0.160255	0.039990	0.040003	0.760792	0.761282	0.760866	0.056	-19.5821	0.0025	0.9927	1.0080
2	25.003	0.981709	0.20	0.160255	0.039990	0.040003	0.758351	0.758964	0.758569	0.056	-19.2602	0.0025	0.9927	1.0067
2	30.004	0.980822	0.20	0.160255	0.039990	0.040003	0.755790	0.756571	0.756191	0.056	-18.9514	0.0025	0.9927	1.0053
2	35.003	0.980451	0.20	0.160255	0.039990	0.040003	0.753117	0.754109	0.753745	0.056	-18.6554	0.0025	0.9927	1.0036
2	40.004	0.979742	0.20	0.160255	0.039990	0.040003	0.750296	0.751567	0.751218	0.056	-18.3712	0.0024	0.9927	1.0017
2	45.009	0.979249	0.20	0.160255	0.039990	0.040003	0.747317	0.748947	0.748615	0.056	-18.0978	0.0024	0.9927	0.9996
2	25.002	0.978601	0.20	0.160255	0.039990	0.040003	0.758376	0.759031	0.758636	0.056	-19.2629	0.0025	0.9927	1.0067
3	25.003	0.980244	0.40	0.360042	0.040003	0.039975	0.744212	0.744842	0.744447	0.056	-19.4026	0.0025	0.9861	1.0147
3	5.013	0.980994	0.40	0.360042	0.040003	0.039975	0.754465	0.754765	0.754348	0.056	-20.7890	0.0028	0.9862	1.0186
3	10.017	0.981107	0.40	0.360042	0.040003	0.039975	0.752075	0.752423	0.751997	0.056	-20.4190	0.0025	0.9862	1.0180
3	15.014	0.981990	0.40	0.360042	0.040003	0.039975	0.749596	0.749998	0.749568	0.056	-20.0656	0.0025	0.9862	1.0171
3	20.007	0.982157	0.40	0.360042	0.040003	0.039975	0.747026	0.747514	0.747098	0.056	-19.7291	0.0025	0.9861	1.0160
3	25.003	0.981709	0.40	0.360042	0.040003	0.039975	0.744351	0.744961	0.744566	0.056	-19.4072	0.0025	0.9861	1.0147
3	30.004	0.980822	0.40	0.360042	0.040003	0.039975	0.741565	0.742342	0.741962	0.056	-19.0987	0.0025	0.9861	1.0131
3	35.003	0.980451	0.40	0.360042	0.040003	0.039975	0.738674	0.739660	0.739296	0.056	-18.8034	0.0025	0.9861	1.0113
3	40.004	0.979742	0.40	0.360042	0.040003	0.039975	0.735645	0.736909	0.736560	0.056	-18.5200	0.0024	0.9861	1.0094
3	45.009	0.979249	0.40	0.360042	0.040003	0.039975	0.732465	0.734085	0.733753	0.056	-18.2478	0.0024	0.9861	1.0073
3	25.002	0.978601	0.40	0.360042	0.040003	0.039975	0.744408	0.745060	0.744665	0.056	-19.4111	0.0025	0.9861	1.0147
4	25.003	0.980244	1.75	1.709978	0.040035	0.039958	0.713532	0.714141	0.713746	0.056	-19.6834	0.0025	0.9399	1.0647

4	5.013	0.980994	1.75	1.709978	0.040035	0.039958	0.726270	0.726564	0.726147	0.056	-21.0883	0.0028	0.9412	1.0713
4	10.017	0.981107	1.75	1.709978	0.040035	0.039958	0.723216	0.723555	0.723129	0.056	-20.7118	0.0025	0.9408	1.0698
4	15.014	0.981990	1.75	1.709978	0.040035	0.039958	0.720097	0.720487	0.720057	0.056	-20.3530	0.0025	0.9404	1.0683
4	20.007	0.982157	1.75	1.709978	0.040035	0.039958	0.716907	0.717379	0.716963	0.056	-20.0120	0.0025	0.9401	1.0666
4	25.003	0.981709	1.75	1.709978	0.040035	0.039958	0.713646	0.714235	0.713840	0.056	-19.6871	0.0025	0.9399	1.0647
4	30.004	0.980822	1.75	1.709978	0.040035	0.039958	0.710296	0.711044	0.710664	0.056	-19.3765	0.0025	0.9397	1.0627
4	35.003	0.980451	1.75	1.709978	0.040035	0.039958	0.706865	0.707813	0.707448	0.056	-19.0798	0.0025	0.9395	1.0606
4	40.004	0.979742	1.75	1.709978	0.040035	0.039958	0.703321	0.704532	0.704183	0.056	-18.7961	0.0024	0.9394	1.0583
4	45.009	0.979249	1.75	1.709978	0.040035	0.039958	0.699652	0.701202	0.700870	0.056	-18.5242	0.0024	0.9393	1.0560
4	25.002	0.978601	1.75	1.709978	0.040035	0.039958	0.713690	0.714321	0.713926	0.056	-19.6905	0.0025	0.9399	1.0647
5	25.003	0.980244	2.00	1.959950	0.040008	0.040012	0.710483	0.711088	0.710693	0.056	-19.6981	0.0025	0.9308	1.0734
5	5.013	0.980994	2.00	1.959950	0.040008	0.040012	0.723490	0.723783	0.723366	0.056	-21.1058	0.0028	0.9323	1.0803
5	10.017	0.981107	2.00	1.959950	0.040008	0.040012	0.720366	0.720703	0.720278	0.056	-20.7284	0.0025	0.9318	1.0788
5	15.014	0.981990	2.00	1.959950	0.040008	0.040012	0.717175	0.717563	0.717133	0.056	-20.3688	0.0025	0.9314	1.0771
5	20.007	0.982157	2.00	1.959950	0.040008	0.040012	0.713926	0.714395	0.713979	0.056	-20.0274	0.0025	0.9310	1.0753
5	25.003	0.981709	2.00	1.959950	0.040008	0.040012	0.710603	0.711188	0.710793	0.056	-19.7020	0.0025	0.9308	1.0734
5	30.004	0.980822	2.00	1.959950	0.040008	0.040012	0.707194	0.707937	0.707557	0.056	-19.3910	0.0025	0.9305	1.0713
5	35.003	0.980451	2.00	1.959950	0.040008	0.040012	0.703708	0.704648	0.704284	0.056	-19.0942	0.0025	0.9303	1.0692
5	40.004	0.979742	2.00	1.959950	0.040008	0.040012	0.700109	0.701310	0.700961	0.056	-18.8101	0.0024	0.9302	1.0669
5	45.009	0.979249	2.00	1.959950	0.040008	0.040012	0.696387	0.697923	0.697591	0.056	-18.5381	0.0024	0.9301	1.0645
5	25.002	0.978601	2.00	1.959950	0.040008	0.040012	0.710644	0.711271	0.710876	0.056	-19.7053	0.0025	0.9308	1.0734
6	25.003	0.980244	2.25	2.210013	0.040005	0.039994	0.707680	0.708280	0.707885	0.056	-19.7067	0.0025	0.9214	1.0819
6	5.013	0.980994	2.25	2.210013	0.040005	0.039994	0.720925	0.721216	0.720799	0.056	-21.1165	0.0028	0.9233	1.0893
6	10.017	0.981107	2.25	2.210013	0.040005	0.039994	0.717748	0.718084	0.717658	0.056	-20.7389	0.0025	0.9227	1.0876
6	15.014	0.981990	2.25	2.210013	0.040005	0.039994	0.714494	0.714880	0.714450	0.056	-20.3785	0.0025	0.9222	1.0858
6	20.007	0.982157	2.25	2.210013	0.040005	0.039994	0.711178	0.711644	0.711228	0.056	-20.0363	0.0025	0.9218	1.0840
6	25.003	0.981709	2.25	2.210013	0.040005	0.039994	0.707801	0.708382	0.707987	0.056	-19.7106	0.0025	0.9214	1.0819
6	30.004	0.980822	2.25	2.210013	0.040005	0.039994	0.704336	0.705073	0.704693	0.056	-19.3992	0.0025	0.9212	1.0798
6	35.003	0.980451	2.25	2.210013	0.040005	0.039994	0.700794	0.701726	0.701362	0.056	-19.1020	0.0025	0.9209	1.0776
6	40.004	0.979742	2.25	2.210013	0.040005	0.039994	0.697147	0.698338	0.697989	0.056	-18.8178	0.0024	0.9208	1.0753
6	45.009	0.979249	2.25	2.210013	0.040005	0.039994	0.693380	0.694902	0.694570	0.056	-18.5457	0.0024	0.9207	1.0728
6	25.002	0.978601	2.25	2.210013	0.040005	0.039994	0.707845	0.708467	0.708072	0.056	-19.7140	0.0025	0.9214	1.0819
7	25.003	0.980244	2.50	2.460012	0.039997	0.040021	0.705149	0.705745	0.705350	0.056	-19.7133	0.0025	0.9119	1.0903
7	5.013	0.980994	2.50	2.460012	0.039997	0.040021	0.718582	0.718872	0.718455	0.056	-21.1241	0.0028	0.9140	1.0980
7	10.017	0.981107	2.50	2.460012	0.039997	0.040021	0.715346	0.715680	0.715254	0.056	-20.7457	0.0025	0.9133	1.0962

7	15.014	0.981990	2.50	2.460012	0.039997	0.040021	0.712049	0.712433	0.712002	0.056	-20.3853	0.0025	0.9128	1.0944
7	20.007	0.982157	2.50	2.460012	0.039997	0.040021	0.708686	0.709148	0.708733	0.056	-20.0429	0.0025	0.9123	1.0924
7	25.003	0.981709	2.50	2.460012	0.039997	0.040021	0.705259	0.705835	0.705440	0.056	-19.7168	0.0025	0.9119	1.0903
7	30.004	0.980822	2.50	2.460012	0.039997	0.040021	0.701750	0.702481	0.702101	0.056	-19.4054	0.0025	0.9116	1.0882
7	35.003	0.980451	2.50	2.460012	0.039997	0.040021	0.698167	0.699091	0.698727	0.056	-19.1081	0.0025	0.9114	1.0859
7	40.004	0.979742	2.50	2.460012	0.039997	0.040021	0.694477	0.695657	0.695308	0.056	-18.8238	0.0024	0.9112	1.0835
7	45.009	0.979249	2.50	2.460012	0.039997	0.040021	0.690670	0.692177	0.691845	0.056	-18.5517	0.0024	0.9110	1.0810
7	25.002	0.978601	2.50	2.460012	0.039997	0.040021	0.705294	0.705912	0.705517	0.056	-19.7199	0.0025	0.9119	1.0903
8	25.003	0.980244	2.75	2.709906	0.039993	0.040082	0.702719	0.703311	0.702916	0.056	-19.7139	0.0025	0.9022	1.0986
8	5.013	0.980994	2.75	2.709906	0.039993	0.040082	0.716229	0.716518	0.716101	0.056	-21.1211	0.0028	0.9046	1.1065
8	10.017	0.981107	2.75	2.709906	0.039993	0.040082	0.712945	0.713277	0.712851	0.056	-20.7425	0.0025	0.9038	1.1047
8	15.014	0.981990	2.75	2.709906	0.039993	0.040082	0.709606	0.709987	0.709557	0.056	-20.3821	0.0025	0.9032	1.1028
8	20.007	0.982157	2.75	2.709906	0.039993	0.040082	0.706209	0.706668	0.706253	0.056	-20.0400	0.0025	0.9026	1.1007
8	25.003	0.981709	2.75	2.709906	0.039993	0.040082	0.702744	0.703316	0.702921	0.056	-19.7141	0.0025	0.9022	1.0986
8	30.004	0.980822	2.75	2.709906	0.039993	0.040082	0.699196	0.699921	0.699541	0.056	-19.4026	0.0025	0.9019	1.0964
8	35.003	0.980451	2.75	2.709906	0.039993	0.040082	0.695579	0.696495	0.696131	0.056	-19.1056	0.0025	0.9016	1.0940
8	40.004	0.979742	2.75	2.709906	0.039993	0.040082	0.691858	0.693027	0.692678	0.056	-18.8216	0.0024	0.9014	1.0916
8	45.009	0.979249	2.75	2.709906	0.039993	0.040082	0.688024	0.689517	0.689185	0.056	-18.5500	0.0024	0.9012	1.0891
8	25.002	0.978601	2.75	2.709906	0.039993	0.040082	0.702726	0.703340	0.702945	0.056	-19.7150	0.0025	0.9022	1.0986
9	25.003	0.980244	3.00	2.960018	0.039999	0.040002	0.700439	0.701026	0.700631	0.056	-19.7120	0.0025	0.8923	1.1067
9	5.013	0.980994	3.00	2.960018	0.039999	0.040002	0.714150	0.714437	0.714020	0.056	-21.1214	0.0028	0.8949	1.1149
9	10.017	0.981107	3.00	2.960018	0.039999	0.040002	0.710814	0.711144	0.710719	0.056	-20.7422	0.0025	0.8940	1.1130
9	15.014	0.981990	3.00	2.960018	0.039999	0.040002	0.707424	0.707803	0.707373	0.056	-20.3812	0.0025	0.8933	1.1110
9	20.007	0.982157	3.00	2.960018	0.039999	0.040002	0.703973	0.704429	0.704014	0.056	-20.0384	0.0025	0.8928	1.1089
9	25.003	0.981709	3.00	2.960018	0.039999	0.040002	0.700458	0.701025	0.700631	0.056	-19.7120	0.0025	0.8923	1.1067
9	30.004	0.980822	3.00	2.960018	0.039999	0.040002	0.696859	0.697578	0.697198	0.056	-19.4000	0.0025	0.8919	1.1044
9	35.003	0.980451	3.00	2.960018	0.039999	0.040002	0.693192	0.694100	0.693736	0.056	-19.1024	0.0025	0.8916	1.1020
9	40.004	0.979742	3.00	2.960018	0.039999	0.040002	0.689427	0.690585	0.690236	0.056	-18.8182	0.0024	0.8914	1.0996
9	45.009	0.979249	3.00	2.960018	0.039999	0.040002	0.685551	0.687029	0.686697	0.056	-18.5463	0.0024	0.8912	1.0971
9	25.002	0.978601	3.00	2.960018	0.039999	0.040002	0.700410	0.701019	0.700624	0.056	-19.7118	0.0025	0.8923	1.1067
10	25.003	0.980244	3.25	3.209959	0.040010	0.039998	0.698222	0.698805	0.698410	0.056	-19.7056	0.0025	0.8822	1.1147
10	5.013	0.980994	3.25	3.209959	0.040010	0.039998	0.712114	0.712400	0.711983	0.056	-21.1164	0.0028	0.8850	1.1232
10	10.017	0.981107	3.25	3.209959	0.040010	0.039998	0.708721	0.709050	0.708624	0.056	-20.7364	0.0025	0.8841	1.1212
10	15.014	0.981990	3.25	3.209959	0.040010	0.039998	0.705286	0.705662	0.705232	0.056	-20.3750	0.0025	0.8833	1.1191
10	20.007	0.982157	3.25	3.209959	0.040010	0.039998	0.701788	0.702241	0.701825	0.056	-20.0318	0.0025	0.8827	1.1169

10	25.003	0.981709	3.25	3.209959	0.040010	0.039998	0.698229	0.698792	0.698397	0.056	-19.7051	0.0025	0.8822	1.1147
10	30.004	0.980822	3.25	3.209959	0.040010	0.039998	0.694591	0.695304	0.694924	0.056	-19.3930	0.0025	0.8818	1.1123
10	35.003	0.980451	3.25	3.209959	0.040010	0.039998	0.690888	0.691788	0.691423	0.056	-19.0954	0.0025	0.8815	1.1099
10	40.004	0.979742	3.25	3.209959	0.040010	0.039998	0.687091	0.688238	0.687889	0.056	-18.8113	0.0024	0.8812	1.1074
10	45.009	0.979249	3.25	3.209959	0.040010	0.039998	0.683187	0.684650	0.684318	0.056	-18.5395	0.0024	0.8811	1.1049
10	25.002	0.978601	3.25	3.209959	0.040010	0.039998	0.698173	0.698778	0.698383	0.056	-19.7046	0.0025	0.8822	1.1147

[a] The meanings of the listed potentials are as follows: E(meas.) – the unadjusted measured values at the listed pressure, P; E – measured potentials corrected to 1 atm pH_2; E(adj.) – values of E adjusted to the standard potentials of Bates and Bower,[17] as noted in section 3 of the main text. [b] The acidity function Q is equal to $\ln(mH^+ \cdot \gamma_{HCl}^2)$, see eq 3 and eq 4.

References

1. Bates, R. G., *Determination of pH, Theory and Practice*, 2nd ed., Wiley: New York, 1973.

2. Maksimov, I.; Asakai, T.; Hibino, Y.; Clegg, S. L., Activity coefficients of HCl in solutions related to 'Tris' Buffers in artificial seawater. I. HCl + TrisHCl + H_2O from 1.0 to 5.0 mol kg^{-1} ionic strength, and from 5 °C to 45 °C. *J. Chem. Eng. Data* **2025,** *70*, 1994-2005.

3. Maksimov, I.; Asakai, T.; Hibino, Y.; Clegg, S. L., Activity coefficients of HCl in solutions related to 'Tris' buffers in artificial seawater. II. HCl + NaCl + TrisHCl + H_2O, and Tris buffer + NaCl + H_2O, to high ionic strength and from 5 °C to 40 °C *J. Chem. Eng. Data* **2025,** *70*, 3614-3629.

4. Bates, R. G.; Robinson, R. A., Standardization of silver-silver chloride electrodes from 0 to 60 °C. *J. Solut. Chem.* **1980,** *9*, 455-456.

5. Holmes, H. F.; Busey, R. H.; Simonson, J. M.; Mesmer, R. E.; Archer, D. G.; Wood, R. H., The enthalpy of dilution of HCl(aq) to 648 K and 40 MPa. Thermodynamic properties. *J. Chem. Thermo.* **1987,** *19*, 863-890.

6. Joint Committee for Guides in Metrology, *Evaluation of measurement data — Guide to the expression of uncertainty in measurement*; BIPM, IEC, IFCC, ILAC, ISO, IUPAC, IUPAP and OIML, 120 pp, doi.org/10.59161/JCGM100-2008E.

7. Bates, R. G.; Guggenheim, E. A.; Harned, H. S.; Ives, D. J. G.; Janz, G. J.; Monk, C. B.; Robinson, R. A.; Stokes, R. H.; Wynne-Jones, W. F. K., Standard electrode potential of the silver, silver chloride electrode. *J. Chem. Phys.* **1956,** *25*, 361.

8. Partanen, J. I.; Covington, A. K., Re-evaluation of the activity coefficients of aqueous hydrochloric acid solutions up to a molality of 2.0 using two-parameter Huckel and Pitzer Equations. Part I. Results at 25 °C. *J. Solut. Chem.* **2002,** *31*, 187-196.

9. Clegg, S. L.; Wexler, A. S., Densities and apparent molar volumes of atmospherically important electrolyte solutions. I. The solutes H_2SO_4, HNO_3, HCl, Na_2SO_4, $NaNO_3$, NaCl, $(NH_4)_2SO_4$, NH_4NO_3, and NH_4Cl from 0 to 50 °C, including extrapolations to very low temperature and to the pure liquid state, and $NaHSO_4$, NaOH and NH_3 at 25 °C. *J. Phys. Chem.* **2011,** *115*, 3393-3460.

10. Ford, T. D.; Call, T. G.; Origlia, M. L.; Stark, M. A.; Woolley, E. M., Apparent molar volumes and apparent molar heat capacities of aqueous 2-amino-2-hydroxymethyl-propan-1,3-diol (Tris or THAM) and THAM plus equimolal HCl. *J. Chem. Thermodyn.* **2000,** *32*, 499-516.

11. Tishchenko, P. Y., Non-ideal properties of the TRIS-TRIS.HCI-NaCI-H_2O buffer system in the 0-40 °C temperature interval. Application of the Pitzer equations. *Russ. Chem. Bull.* **2000,** *49* (4), 674-679.

12. Archer, D. G., Thermodynamic properties of the NaCl + H_2O system II. Thermodynamic properties of NaCl(aq), NaCl.2H_2O(cr), and phase equilibria. *J. Phys. Chem. Ref. Data* **1992,** *21*, 793-829.

13. Millero, F. J.; Hershey, J. P.; Fernandez, M., The pK* of $TRISH^+$ in Na-K-Mg-Ca-Cl-SO_4 brines - pH scales. *Geochim. et Cosmochim. Acta* **1987,** *51*, 707-711.

14. Lodeiro, P.; Turner, D. R.; Achterberg, E. P.; Gregson, F. K. A.; Reid, J. P.; Clegg, S. L., Solid−liquid equilibria in aqueous solutions of Tris, Tris-NaCl, Tris-TrisHCl, and Tris-$(TrisH)_2SO_4$ at temperatures from 5 to 45 °C. *J. Chem. Eng. Data* **2021,** *66* (437-455).

15. Bates, R. G.; Macaskill, J. B., Activity and osmotic coefficients of t-butylammonium chloride: activity of HCl in mixtures with TRIS hydrochloride and t-butylammonium chloride at 25°C. *J. Solut. Chem.* **1985,** *14*, 723-734.

16. Harvie, C. E.; Weare, J. H., The prediction of mineral solubilities in natural waters: the Na-K-Mg-Ca-Cl-SO_4-H_2O systems from zero to high concentration at 25 C. *Geochim. et Cosmochim. Acta* **1980,** *44*, 981-997.

17. Bates, R. G.; Bower, V. E., Standard potential of the silver-silver chloride electrode from 0 to 95 °C and the thermodynamic properties of dilute hydrochloric acid solutions. *J. Res. Natl. Bur. Stnds.* **1954,** *53*, 283-290.
